# Advances and Perspectives of H_2_ Production from NH_3_ Decomposition in Membrane Reactors

**DOI:** 10.1021/acs.energyfuels.3c00760

**Published:** 2023-07-16

**Authors:** Valentina Cechetto, Luca Di Felice, Fausto Gallucci

**Affiliations:** † Inorganic Membranes and Membrane Reactors, Sustainable Process Engineering, Department of Chemical Engineering and Chemistry, Eindhoven University of Technology, De Rondom 70, 5612 AP Eindhoven, The Netherlands; ‡ Eindhoven Institute for Renewable Energy Systems (EIRES), Eindhoven University of Technology, P.O. Box 513, 5600 MB Eindhoven, The Netherlands

## Abstract

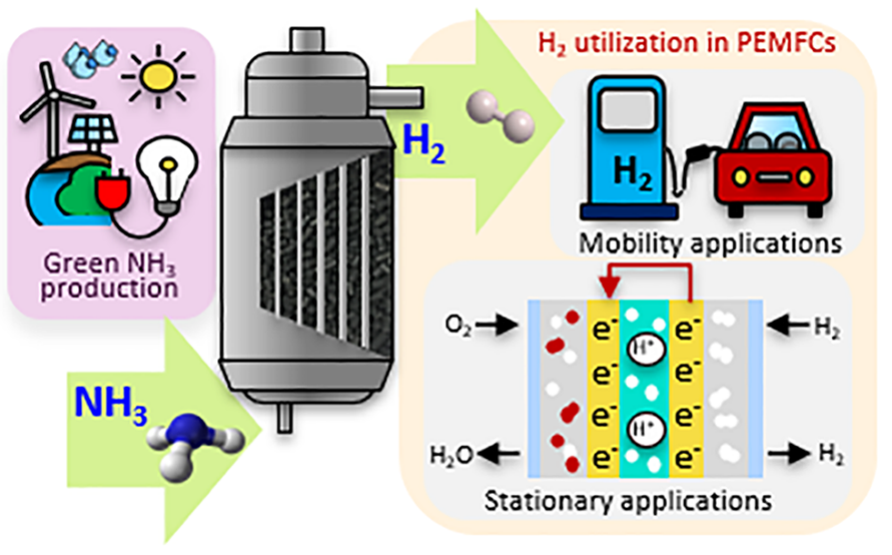

Hydrogen is often regarded as an ideal energy carrier. Its use in energy conversion devices does in fact not produce any pollutants. However, due to challenges related to its transportation and storage, liquid hydrogen carriers are being investigated. Among the liquid hydrogen carriers, ammonia is considered very promising because it is easy to store and transport, and its conversion to hydrogen has only nitrogen as a byproduct. This work focuses on a review of the latest results of studies dealing with ammonia decomposition for hydrogen production. After a general introduction to the topic, this review specifically focuses on works presenting results of membrane reactors for ammonia decomposition, particularly describing the different reactor configurations and operating conditions, membrane properties, catalysts, and purification steps that are required to achieve pure hydrogen for fuel cell applications.

## 1 Introduction

The observed climate changes due to anthropogenic CO_2_ emissions, cost of energy, and energy security are key challenges that today’s society is facing.^1−3^ The depletion of fossil fuels combined with the urgency to mitigate global warming and reduce the negative environmental impact of a fossil fuel-based energy system is in fact motivating a transition toward a new, cleaner, and more efficient energy scenario.^2,4,5^ The exploitation of renewable energy sources for power production plays a fundamental role in the energy transition, but the intermittent nature of energy resources represents a challenge for the stability of the electricity grid that must be adequately addressed.^6−8^ While the scientific community agrees that energy storage is undoubtedly the key to overcome this issue and increase the share of renewable energy sources in their generation capacity,^1^ the fact that large wind and photovoltaic power plants are often located far away from the consumption site suggests that large quantities of renewable energy should be stored in the form of dispatchable energy carriers.^4^

Over the last decades, hydrogen has gained attention as a viable future replacement for fossil fuels and as the ideal energy carrier.^9−13^ Not only can green hydrogen be produced by exploiting surplus (renewable) power for water electrolysis serving as storage media for renewable electricity, but having higher energy density compared to conventional fuels and being carbon neutral, it could be used as fuel for clean power production.^14,15^ Nevertheless, the commercialization of hydrogen-based technologies at industrial scale is hampered by challenges related to hydrogen transportation and long-term storage. Particularly, the low volumetric energy density and the low boiling point of hydrogen require both high pressures and low temperatures for practical storage and transportation.^7^ The widespread use of hydrogen-based technologies on an industrial scale therefore requires the infrastructure for hydrogen supply to be improved. A possible solution suggested for this challenge consists in storing hydrogen energy in the chemical bonds of hydrogen carrier compounds.^16^ Liquid fuels generated from hydrogen could in fact be easily liquefied, transported over long distances, and finally either used for particular applications requiring them as feedstock or decomposed to produce hydrogen when required.^17,18^ Several liquid fuels have been reported in the literature as potential media for hydrogen storage,^19−21^ and among all, ammonia stands out due to its numerous advantages compared to both hydrogen and other possible hydrogen carriers.^20,22,23^ Most importantly, its ease of liquefaction compared to compressed hydrogen and its lower cost per unity of energy stored (0.54 $/kg_H2_ for ammonia and 14.95 $/kg_H2_ for hydrogen^1^), as well as its already existing infrastructure for storage and transportation, allow for economically competitive and relatively easy and safe hydrogen storage and transportation. Second, the fact that ammonia is a carbon-free molecule makes it attractive for several applications including its direct use for power generation and its use as a hydrogen vector.^1,4,14,24−30^ All in all, ammonia can be regarded as an ideal hydrogen carrier and is expected to be one of the major contributors to a carbon-free economy.

**Figure 1:**
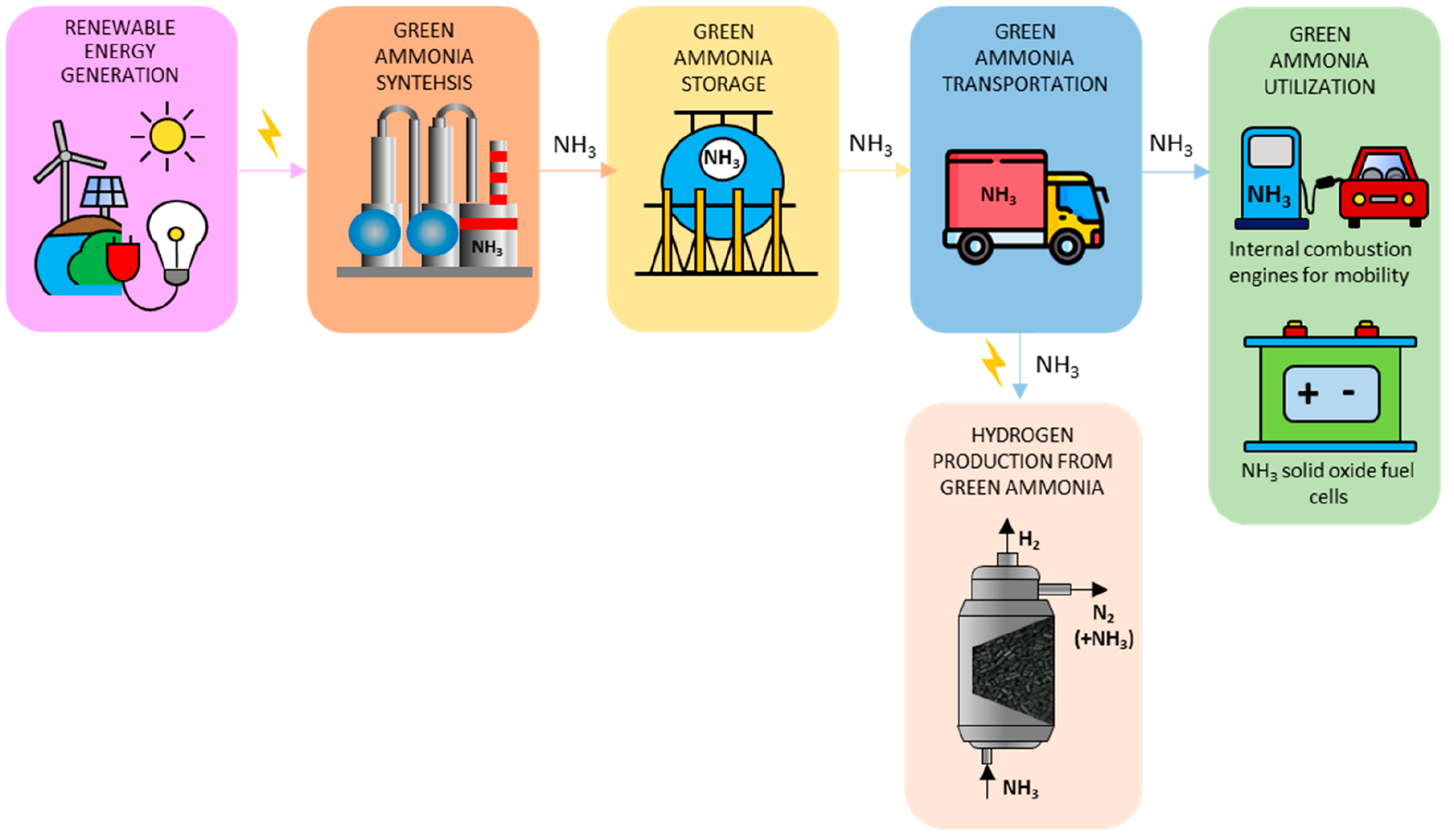
Value chain of production, distribution, and utilization of green ammonia.

A schematic representation of the entire value chain of green ammonia production, distribution, and utilization is depicted in Figure 1. In this value chain, ammonia decomposition at large scale is the most technically challenging step,^7^ and at the moment, there are no publicly known large-scale units for ammonia decomposition able to deliver hundreds of tons of hydrogen per day in a single production train.^31^ The release of hydrogen from ammonia is in fact an energy intensive process; thus, high energy efficiencies in the utilization of ammonia are hard to be achieved but required for its applicability. To the best of our knowledge, ThyssenKrupp is the only group of companies in the world able to offer the entire hydrogen value chain from water electrolysis through ammonia production and storage to ammonia cracking.^32^

From a technical point of view, hydrogen production from ammonia (NH_3_) consists of two steps: ammonia decomposition into hydrogen (H_2_) and nitrogen (N_2_) and hydrogen separation. As one of the limitations inhibiting the widespread use of this technology at larger scale is the development of reliable, efficient, and scalable processes integrating the ammonia decomposition reactor with hydrogen purification systems,^33^ in this work, state-of-the-art literature about hydrogen production from ammonia decomposition is explored with a focus on membrane reactor technology for high purity hydrogen production. This technology allows in fact for two main advantages over conventional systems. First, both the ammonia decomposition reaction and hydrogen separation are integrated into one single step. Second, a shift in the ammonia decomposition reaction equilibrium results in the achievement of ammonia conversions comparable to those obtained in conventional systems at lower temperatures.^34^ This high level of process intensification can lead to substantial benefits in terms of process efficiency.

Moreover, while hydrogen recovered from ammonia could be used in a wide range of possible applications, such as for example hydrogen refueling stations, the carbon-free nature of ammonia makes it particularly attractive for the production of hydrogen to be used as fuel in proton exchange membrane fuel cells (PEMFCs). When hydrogen for powering the fuel cell is produced via ammonia decomposition, the risk of carbon poisoning of the cell electrodes is in fact circumvented. We therefore choose to focus this work on reviewing the state-of-the-art literature on ammonia decomposition and in particular on membrane reactors for the production of pure hydrogen to be specifically used as fuel for power production in PEMFCs. Furthermore, since PEMFC specifications impose that ammonia concentrations in the hydrogen stream used as feedstock must be lower than 0.1 ppm^35^ to prevent the deactivation of anode catalyst and the consequent decrease in the cell performance, particular attention is paid to those works in which the target purity of hydrogen for fuel cell application was considered and/or achieved.

### 1.1 Ammonia as Hydrogen Carrier

Ammonia is an inorganic compound of nitrogen (83.2 wt %) and hydrogen (17.8 wt %), which is alkaline, corrosive, and colorless and has a distinct pungent smell. Its primary use is nowadays dedicated to the fertilizer industry, but due to its physical properties, it has also recently been regarded as a valuable alternative for hydrogen storage and transportation. A comparison between the characteristics of hydrogen storage in its pure form and in the chemical form of ammonia is presented in Table 1. While hydrogen storage is technically possible only at high pressure or low temperature, ammonia can be stored in its liquid form at mild pressure, namely, 9.9 bar at 25 °C, and, therefore, its storage and transport are relatively easier and less energy intensive compared to hydrogen. Moreover, ammonia has both higher volumetric energy density and volumetric hydrogen content compared to hydrogen.

**Table 1:** Physical Properties of Compressed Hydrogen, Liquid Hydrogen and Ammonia for Hydrogen Storage^7,36,38^

Property	Compressed hydrogen	Liquid hydrogen	Liquid Ammonia
Storage method	Compression	Liquefaction	Liquefaction
Storage temperature [°C]	25 (room)	–252.9	25 (room)
Storage pressure [bar]	690	1	9.9
Density [kg/m^3^]	39	70.8	600
Explosive limit in air [vol %]	4–75	4–75	15–28
Gravimetric energy density (LHV) [MJ/kg]	120	120	18.6
Volumetric energy density [MJ/L]	4.5	8.49	12.7
Gravimetric hydrogen content [wt %]	100	100	17.8
Volumetric hydrogen content [kg_H2_/m^3^]	42.2	70.8	121
Gaseous hydrogen production method	Pressure release	Evaporation	NH_3_ decomposition
Energy required for gaseous hydrogen extraction [kJ/mol_H2_]	–	0.907	30.6

From a safety point of view, ammonia has a higher autoignition temperature (650 °C) compared to hydrogen (520 °C) and therefore has a lower risk of fire. Moreover, due to its narrow flammability range, which is 15.15%–27.35% in dry air and 15.95%–26.55% in 100% relative humidity air, ammonia is regarded as non-flammable during storage and transportation. The risk of fire and explosion in case of leakage from a storage vessel is also minimized by the fact that by having a lower density compared to air, ammonia can dissipate quickly in atmosphere.

Hydrogen storage in the form of ammonia has also some disadvantages and challenges that deserve to be carefully analyzed. First, while the regassification of liquid hydrogen only requires 0.907 kJ/mol_H2_, hydrogen production from ammonia requires 30.6 kJ/mol_H2_. This is due to the fact that being ammonia decomposition an endothermic process, energy needs to be supplied in order to promote the reaction. Moreover, while compressed liquid hydrogen can deliver extremely pure hydrogen, the production of high purity hydrogen from ammonia includes two/three stages, namely, ammonia decomposition and hydrogen separation/purification. Finally, when handling ammonia, accurate hazard management will have to be carried out in order to ensure safe handling and utilization as well as to mitigate potential danger to humanity and environment.^36^ Ammonia is in fact categorized as a toxic chemical as it can lead to severe consequences for human health depending on the route, dose, and duration of exposure.^37^

### 1.2 Ammonia Decomposition: Thermodynamic Considerations

NH_3_ decomposition into H_2_ and N_2_ occurs according to the following reaction (eq 1):
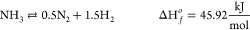
1

**Figure 2:**
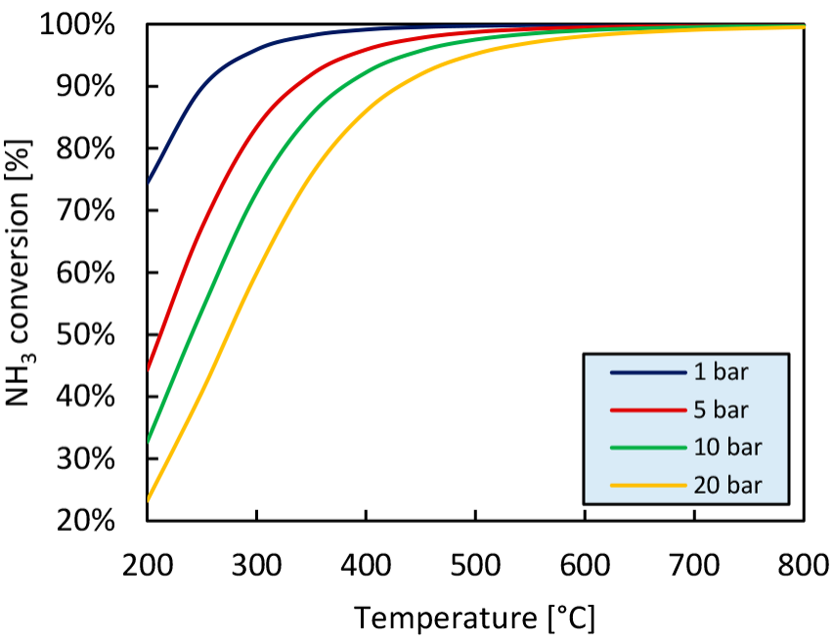
NH_3_ equilibrium conversion as a function of temperature for different pressures.

The reaction is mildly endothermic and is therefore thermodynamically favored at high temperatures. Moreover, according to the Le Châtelier’s principle, as ammonia decomposition occurs with molar expansion, it is favored at low pressure. In Figure 2, the equilibrium conversion is reported as a function of temperature and pressure. The values of conversions of NH_3_ into H_2_ were calculated using Aspen Plus V11 software and using a Gibbs reactor (free energy minimization method). Figure 2 shows that ammonia conversion is favored by low pressure and high temperature, achieving >99% at 1 bar and *T* > 400 °C. Above 500 °C, the thermodynamic conversion is >95% in the whole pressure range investigated.

### 1.3 Focus of This Review

The aim of this work is to review the most critical aspects of ammonia decomposition through the innovative approach of membrane reactors for one-step (enhanced) ammonia decomposition and hydrogen separation. First, a simplified schematic view of ammonia cracking at a system level is given to highlight the process intensification approach of the membrane reactor as compared to the benchmark conventional technology. Then, focus is given on encompassing literature data on the building blocks of the membrane reactor configuration, namely, the cracking catalyst (where, after a brief overview, the reader is redirected to an already existing recent review paper) and the type of H_2_-selective membranes that can be suitable for this purpose. Subsequently, the possible reactor configurations, the comparison of performances of such membrane reactors based on the existing data, and the effects of operating conditions are encompassed, followed by the identification of the most “efficient” membrane reactors currently documented in the literature. The critical point of hydrogen cleaning from residual ammonia traces is discussed. Finally, the cracking of ammonia from diluted streams is also addressed for the sake of a complete overview on the efforts perfused on catalytic ammonia decomposition in the presence of a membrane reactor.

## 2 Hydrogen Production from Ammonia Decomposition with and without Membranes: A Schematic View

**Figure 3:**
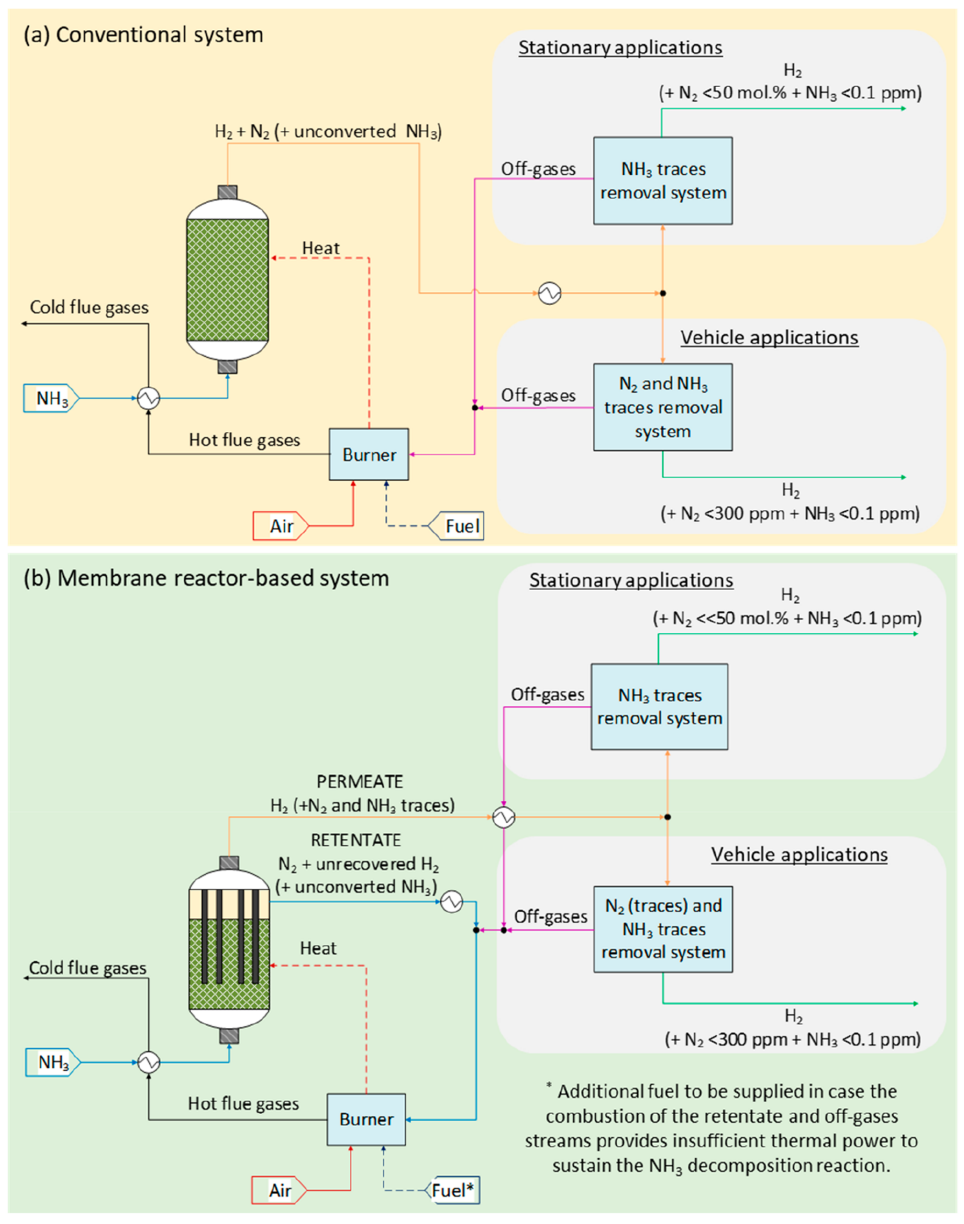
Schematic representation of a conventional system (a) and a membrane reactor-based system (b) for hydrogen production via ammonia decomposition.

Hydrogen production and recovery from ammonia decomposition require a reactor unit operating at high temperature to favor ammonia conversion and a separation system to extract pure hydrogen from nitrogen and unconverted ammonia (Figure 3).

In a conventional system (Figure 3(a)), the produced hydrogen is diluted with nitrogen and contains the unconverted ammonia exiting the cracker. The separation of hydrogen from nitrogen and residual ammonia traces can subsequently take place by means of different techniques. Pressure swing adsorption (PSA) is the most well developed technology for the separation of hydrogen produced via ammonia decomposition,^23^ but given the impracticality and safety complications of large quantities of hydrogen in storage vessels in pressurized conditions, cryogenic separation can be considered as a valid alternative, especially for large scale applications.^39^ For hydrogen purification from ammonia, instead, given the high solubility of ammonia in water, the unreacted ammonia can be removed through water absorption. Alternatively, commercially available adsorbent materials^24,28,40−44^ and ion-exchange forms of different type of zeolites^44−47^ can also be used to reduce the residual ammonia concentration in the hydrogen stream to levels that are suitable for PEM fuel cell application through adsorption. As another alternative, RenCat proposed a selective ammonia oxidation reactor (SAO) as a cleaning method,^48^ but data on catalyst performance and durability have not been found in the literature.

However, despite the relative low complexity of the process, one of the limitations inhibiting the widespread use at larger scales of viable conventional energy systems based on ammonia as the energy carrier is the development of efficient and scalable processes for hydrogen recovery integrating the ammonia decomposition reactor with the hydrogen separation systems.^33^ In this regard, a good solution is offered by membrane reactor technology (Figure 3(b)), in which the NH_3_ decomposition reaction and the selective separation of hydrogen are simultaneously performed in a single unit. By selectively separating one of the reaction products (in this case hydrogen), the thermodynamic limitations of conventional systems are circumvented and, as a result, the membrane system can achieve comparable or higher conversion compared to the conventional technology while operating at lower temperatures and with a more compact design. This brings benefits both from energy efficiency and economic points of view. In the (H_2_-selective) membrane reactor, H_2_/N_2_ separation is inherent into the cracker. However, a purification stage for nitrogen (from few percentages to traces depending on the type of membrane used) and/or ammonia trace removal might still be required depending on the membrane properties and end use of hydrogen.

According to the standards available for the composition requirement of hydrogen for PEMFC application (ISO 14687:2019^35^), N_2_ concentration can constitute up to 50% of the feed gas by volume in stationary applications and must not exceed 300 ppm in vehicle applications. NH_3_ concentration must instead not exceed 0.1 ppm in both applications. From the stoichiometry of the ammonia decomposition reaction, it follows that in plants for the production of hydrogen to be used in stationary applications a nitrogen separation unit is not needed, neither in a conventional system nor in a membrane reactor-based system. Conversely, in the case of plants for the production of hydrogen to be used in vehicle applications, a nitrogen separation unit is always required in conventional systems, while it may not be necessary in membrane reactor-based systems if the H_2_/N_2_ selectivity of the membranes is sufficiently high. The very low limits imposed on the residual concentration of ammonia in hydrogen, on the other hand, require the use of an ammonia separation unit in all the scenarios.

Given the endothermicity of the ammonia decomposition reaction, both in conventional and in membrane reactor-based systems, heat must be supplied to the reaction unit to thermally sustain the ammonia decomposition reaction. The off-gases leaving the hydrogen purification units can be used as fuel for the generation of such heat. Additional fuel is however required, as the solely combustion of off-gases provides insufficient input. The direct combustion of ammonia itself for heat generation is economically favorable. However, due to its narrow flammability limit, in configurations in which it would be mixed with off-gases highly diluted in nitrogen (such as for example in the case of a conventional plant for the production of hydrogen for vehicle applications), it might be required to combust also a fraction of the produced hydrogen in order to ensure a formulation of the combustible mixture within flammability limits. In addition, in a membrane reactor-based system, the retentate stream containing unconverted ammonia and unrecovered hydrogen can also be used as fuel to supply the heat necessary to thermally sustain the endothermic cracking reaction. This combination can be used to prevent the use of extra (decarbonized) fuel for green ammonia production.

## 3 Catalysts for Ammonia Decomposition

Catalytic ammonia decomposition reactions occur at lower reaction temperature than the equivalent non-catalytic thermal process, and even lower temperatures are targeted for an efficient use of a membrane reactor. Hence, the choice of the most suitable catalyst has a key role in reducing the energy consumption as well as in improving the process safety.

Following the principle of microreversibility in heterogeneous catalysis, early studies on catalysis for ammonia decomposition considered Ru and Fe,^38^ which are well known to catalyze ammonia synthesis in the Haber–Bosch process. Afterward, Cu-based^49^ catalysts were investigated as well as other single (Ni, Ir, Mo, Co, Pt, Pd, and Rh^50^), dual (Co-Mo, Ni-Mo, Fe-Mo, Ni-Co, Co-Mo-Fe-Ni-Cu, Mg-Fe, Fe-Co, Ni-Fe, Mg-Co-Fe, Ni-Pt, Ni-Pd, Ir-Ni, Cu-Zn), and bimetallic active phases including Ru.^51,52^ Ganley et al.^53^ investigated several metals as possible catalysts for ammonia decomposition supported on alumina and identified their catalytic activity to be Ru > Ni > Rh > Co > Ir > Fe ≫ Pt > Cr > Pd > Cu ≫ Te, Se, Pb. Different supports have also been demonstrated to have an influence on the catalytic activity of the material.^52,54,55^ Nonetheless, all the studies available in the literature agree on the fact that the most active metal for ammonia decomposition is ruthenium^10,52,54^ supported on different types of materials, including activated carbon,^56^ metal oxides,^57^ carbon nanotubes,^58^ alumina,^51,59^ and mesoporous silica.^60,61^ Recent studies on innovative Ru-based catalyst formulation are focusing on further reducing the ammonia decomposition reaction temperature below 450 °C.^50^ Nevertheless, since ruthenium is an expensive, rare noble metal with high environmental impact and energy demanding extraction, its replacement with low-cost alternatives is desired.^50,52,62^ For a thorough discussion on the topic of catalysis for ammonia decomposition, we refer the reader to the recent review article by Lucentini et al.^52^

## 4 Membrane Reactors for Hydrogen Production from Ammonia Decomposition

### 4.1 Membranes for Hydrogen Separation during Ammonia Decomposition in Membrane Reactors: Formulation and Reactor Configuration

#### 4.1.1 Selective Layer

An overview of the different types of membranes that have been used in the literature for hydrogen separation during ammonia decomposition in membrane reactors is given in Table 2. Pd-based membranes are the most commonly used, and this is due to multiple aspects: (1) Compared to other types of membranes, they show outstanding performance in terms of both high permeance and high selectivity toward hydrogen. (2) They have already been widely studied for hydrogen separation in several processes^63^ and can be therefore considered as a mature technology. (3) They have been shown to be stable in the presence of ammonia.^64^ In the works of Cechetto et al.,^40,65^ Cerrillo et al.,^66^ Liu et al.,^67^ and Jiang et al., Pd-alloy membranes were selected for hydrogen separation. Compared to pure Pd, they showed higher permeation rates of hydrogen due to the higher diffusivity of atomic hydrogen compared to pure Pd.^69^ Moreover, while Pd membranes suffer from hydrogen embrittlement at temperatures and pressures lower than 300 °C and 2 MPa, respectively,^70−72^ as well as from the formation of defects causing a reduction in perm-selectivity at temperatures higher than 550 °C,^73,74^ Pd-alloy membranes show higher thermal and chemical stability.

**Table 2:** Overview of Different Types of Membranes Used in Literature for Hydrogen Separation in Membrane Reactors for Ammonia Decomposition

	Membrane				
Author(s) [ref]	Selective layer composition	Selective layer thickness [μm]	Membrane configuration	Type of support	Support material (thickness)
Zhang et al.^17^	Pd	6.2	Supported tubular conventional and catalytic Pd membrane	Ceramic	YSZ (130 mm)
Zhang et al.^27^	Pd	∼3	Supported tubular Pd membrane	Ceramic	Al_2_O_3_ (N/A)
Cechetto et al.^40^	Pd-Ag	∼6–8	Supported tubular Pd-based membrane with a porous Al_2_O_3_-YSZ protective layer	Ceramic	α-Al_2_O_3_ (3.5 mm)
Cechetto et al.^65^	Pd-Ag	4.61	Supported tubular Pd-based membrane with a porous Al_2_O_3_-YSZ protective layer	Ceramic	α-Al_2_O_3_ (2 mm)
Cerrillo et al.^66^	Pd-Au	8	Supported tubular Pd-based membrane	Ceramic	N/A
Liu et al.^67^	Pd/Pd-Ag	6.5–8.1	Supported tubular Pd-based membranes	1) Metallic	1) Stainless steel (+MnO_*x*_)
				2) Ceramic	2) Al_2_O_3_ (+MnO_*x*_)
Li et al.^25,76,88^	SiO_2_	<0.3	Tubular silica membrane on a bimodal catalytic support	Ceramic	Ru/γ-Al_2_O_3_/α-Al_2_O_3_ (1 mm)
Jiang et al.^77^	1) MFI zeolite modified with catalytic cracking deposition of methyldiethoxysilane	1) ∼8.2	1) Supported hollow fiber membrane	Ceramic	1) Al_2_O_3_ (1.5 mm)
	2) Pd-Ag	2) ∼1.8	2) Supported tubular Pd-Ag		2) N/A
	3) CMSM	3) ∼0.9	3) Supported tubular CMSM		3) N/A
Itoh et al.^79^	Pd	200	Tubular Pd membrane	Unsupported membrane	N/A
Itoh et al.^80^	1) Pd	1) 2	1) Supported tubular Pd membrane	1) Ceramic	1) α-Al_2_O_3_ (N/A)
	2) Pd-Ag	2) 200	2) Tubular Pd-Ag membrane	2) Unsupported membrane	2) N/A
Kim et al.^82^	Pd	∼5	Supported tubular Pd membrane	Metallic	Inconel 600 (N/A)
Omata et al.^83^	Pd-Ag/V-Fe	∼0.2 μm Pd-Ag	Supported tubular Pd-based membrane	Metallic	V-10 mol %-Fe alloy (∼100 μm V-Fe)
		∼100 μm V-Fe			
Jo et al.^84^	Pd/Ta	∼0.4 μm Pd	Supported tubular Pd-based membrane	Metallic	Tantalum (∼250 μm)
		∼250 μm Ta			
Park et al.^85^	Pd/Ta/Pd	∼1–2 μm Pd	Supported tubular Pd-based membrane	Metallic	Tantalum (∼250 μm)
		∼250 μm Ta			
Israni et al.^86^	Pd	∼4	Supported hollow fiber Pd nanopore membrane	Ceramic	Pd/γ-Al_2_O_3_/α-Al_2_O_3_
Israni et al.^86^	Pd	∼13	Supported hollow fiber Pd membrane	Ceramic	α-Al_2_O_3_ (0.5 mm)
Sitar et al.^87^	Pd	4.23	Supported tubular catalytic and noncatalytic Pd membrane	Ceramic	YSZ (130 mm)
Rizzuto et al.^89^	Pd	N/A	Supported tubular Pd membrane	Metallic	N/A

Conventional Pd and Pd-alloy membranes are generally prepared by deposition of a layer of Pd or Pd alloy on top of a support. When immersed in a catalyst bed, the selective layer of these membranes is in direct contact with the catalyst particles and can be therefore subject to damage due to friction or chemical interaction between the membrane surface and the catalyst particles. In order to mitigate this effect, Cechetto et al.^40,65^ performed hydrogen separation by means of Pd-based double-skinned membranes. This type of membrane shows outstanding performance in terms of permeance and hydrogen selectivity while having over their selective layer a mesoporous Al_2_O_3_-YSZ layer with thickness of ∼1 μm which has the function to protect the membrane surface.^63,75^

Since one of the major disadvantages of Pd-based membranes is their high production cost, Li et al.^76^ suggested the use of silica membranes. However, their low hydrogen selectivity requires the addition of a unit downstream from the reactor to remove both nitrogen and residual ammonia from the permeate.

A comparison between the performance of different types of membranes for hydrogen separation during ammonia decomposition was carried out by Jiang et al.^77^ employing a modified MFI zeolite membrane, a carbon molecular sieve membrane (CMSM), and a Pd-Ag membrane. The results of this study confirmed Pd-based membranes to be the most selective toward hydrogen compared to MFI zeolite membranes and CMSMs.

Membranes with different thicknesses of the selective layer have been used in the literature as shown in Table 2. In general, the thicker the membrane selective layer is, the fewer the membrane defects are, and the lower then the impurities content in the produced hydrogen, which results in higher membrane selectivity; at the same time, the thicker the selective layer is, the lower the hydrogen permeation is through the membrane wall, which results in lower hydrogen permeance.^40^ In other words, the use of thick membranes can be beneficial to target high hydrogen purities, albeit this comes at the expenses of lower hydrogen recoveries per unit area of the membrane as well as higher costs for the preparation of the membrane.

#### 4.1.2 Supports

Dense Pd-based membranes can be classified into two main groups: unsupported membranes and supported membranes.^78^ As shown in Table 2, both types have been investigated in the literature for hydrogen separation during ammonia decomposition. Unsupported membranes are made of a relatively thick layer of Pd or Pd alloy.^79,80^ While providing good mechanical stability, their relatively thick selective layer hinders hydrogen permeation and results in increased costs for membrane fabrication. On the other hand, ultrathin supported membranes are generally preferred as they incorporate a thin, less expensive selective Pd or Pd-alloy layer on the surface of a porous material that provides the required mechanical resistance to the membrane.

The support materials can be divided into two categories, namely, ceramic and metallic, each one having its own advantages and disadvantages. Particularly, metallic supports ensure good mechanical properties while having thermal expansion coefficients similar to the one of palladium and therefore lowering the risk of crack formation at high temperature at the Pd/support interface. Moreover, membranes with metallic supports are easily sealed and coupled to stainless-steel reactors modules,^78^ which facilitate their integration in industrial applications. Metallic supports, however, present relatively large pores with a wide pore size distribution which makes the generation of a thin and defect-free Pd layer difficult. Moreover, as metal interdiffusion between a support and Pd-based selective layer might take place after operating the membrane at high temperature for long times, a metallic supported membrane might suffer from a marked decrease in the permeation capacity overtime.^78^ These two major disadvantages of metallic supports can be overcome by ceramic supports. The smoother surfaces of ceramic supports with accurate control on porosity and narrow pore size distributions up to a few nanometers^81^ facilitate in fact the deposition of ultrathin and defect-free palladium layers. On the other hand, ceramic materials present a thermal expansion coefficient significantly different to that of palladium as well as lower mechanical resistance compared to metallic supports.

All in all, the selection of either one or the other type of support depends on whether the main objective is to ensure the incorporation of an ultrathin Pd-based layer without defects or to facilitate the integration of the membrane in a reactor module.^78^ In the specific field of hydrogen production from ammonia, Inconel,^82^ stainless-steel,^67^ V-Fe alloy,^83^ and tantalum^84,85^ have been used in the literature as metallic supports for Pd-based membranes, while alumina (Al_2_O_3_)^27,40,65,67,77,80,86^ and YSZ^17,87^ have been used as ceramic supports. Ceramic supports were also used for the fabrication of silica membranes in the works of Li et al.^25,76,88^ as well as for the preparation of CMSM- and MFI-modified zeolites membranes in the work of Jiang et al.^77^ More details on the composition of the supports used in each work can be found in Table 2.

Interestingly, besides providing mechanical stability to the membrane, some supports show also perm-selective properties toward hydrogen. Park et al.^85^ and Jo et al.,^84^ for instance, used tantalum tubes with 250 μm thicknesses, and Omata et al.^83^ used a vanadium membrane with a 100 μm thickness as supports. Due to their inherent selectivity toward hydrogen diffusion,^34^ these supports allow one to achieve good overall membrane separation performance with the addition of a thin Pd layer. However, a disadvantage of this solution is that these types of supports are more costly compared to other support materials, such as alumina and yttria-stabilized zirconia (YSZ), and may still need a small layer of Pd for hydrogen splitting.

Lastly, innovative membrane designs have also been explored. Next to the use of a conventional Pd membrane on a ceramic support, Israni et al.^86^ proposed the use of novel membranes in which a thin layer of Pd is grown within the pores of a supported nanoporous layer. The membrane synthesis was carried out according to the following steps: (1) A layer of γ-Al_2_O_3_ was deposited onto the surface of a hollow fiber α-Al_2_O_3_ support followed by a Pd sensitization/nucleation step. (2) Another γ-Al_2_O_3_ layer was deposited on the nucleated surface. (3) Finally, a Pd layer was deposited. This procedure ensured that because of the smaller pore size and smoother surface of γ-Al_2_O_3_ compared to α-Al_2_O_3_, Pd deposition via electroless plating was facilitated resulting in the fabrication of a ultrathin and defect-free palladium layer. This membrane configuration has been shown to be more resilient to further defect formation during high temperature operation than conventional Pd membranes as well as to allow the reduction of membrane selective layer thickness, while guaranteeing good hydrogen permeation flux and perm-selectivity.

#### 4.1.3 Membrane Reactor Configuration

**Figure 4:**
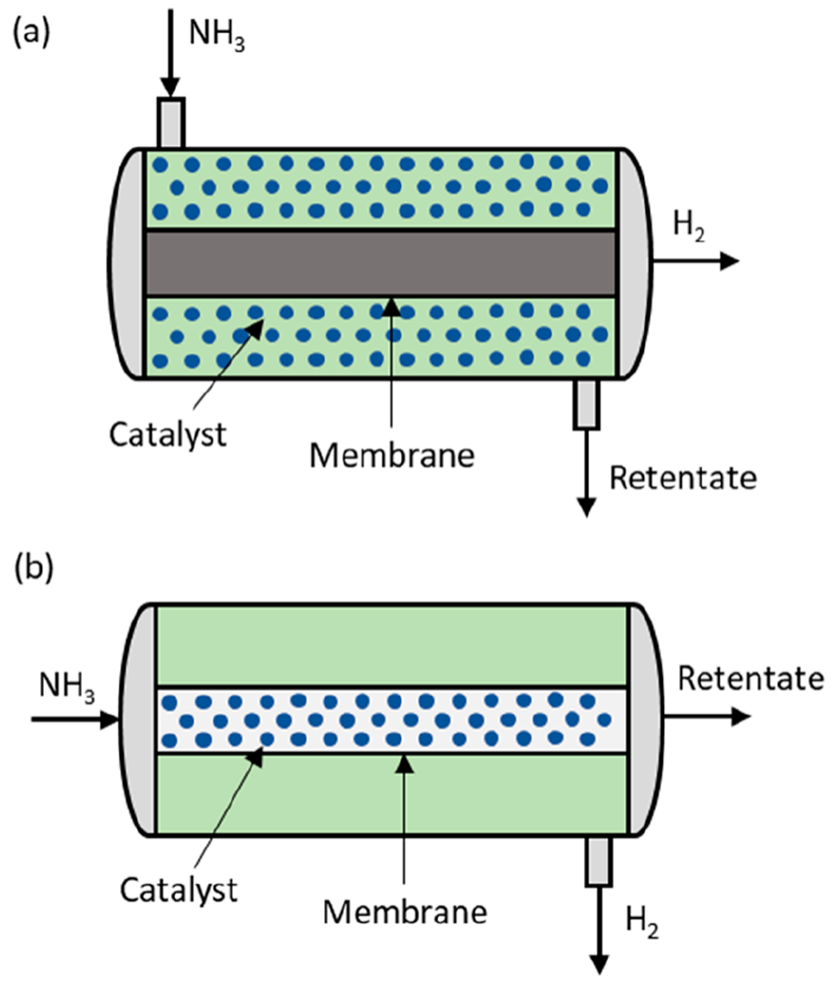
Packed-bed membrane reactor: shell (a) and tube (b) configurations.

In most of the works, the membrane reactor for ammonia decomposition is a packed-bed membrane reactor. In this configuration, the catalytic fixed-bed promotes an ammonia decomposition reaction, and the membrane—which is catalytically inert and linked to the catalyst bed—acts as a pure hydrogen separator. The typical arrangement is a tubular one (Figure 4), in which the catalyst can be situated either in the shell side of the reactor or in the membrane tube, and hydrogen is recovered on the opposite side of the membrane. While this is the most commonly used membrane reactor configuration, Zhang et al.,^17^ Sitar et al.,^87^ and Li et al.^76^ proposed the use of an ammonia decomposition membrane reactor in which the catalyst is impregnated into the membrane support. Since in this configuration both catalytic reaction and gas separation are accomplished by means of the membranes and it is therefore possible to avoid the use of a catalytic bed, this reactor configuration has an advantage in terms of compactness but may result in low mechanical stability at larger transmembrane pressure gradients.

### 4.2 Performance of Conventional and Membrane Reactors: A Comparison

As previously mentioned, one of the advantages of a membrane reactor for hydrogen production from ammonia decomposition over a conventional system consists of the fact that the continuous selective removal of hydrogen from the reaction zone enhances the reaction kinetics and shifts the reaction equilibrium toward the reaction products thereby increasing the conversion of the feedstock. As a result, a membrane reactor allows one to obtain conversions comparable or higher than those of a conventional reactor in smaller volumes and at lower temperatures, with benefits in terms of compactness as well as in terms of energy efficiency. A direct comparison between the performance of the membrane and conventional reactors for hydrogen production from ammonia decomposition is presented in this section based on experimental evidence.

**Figure 5:**
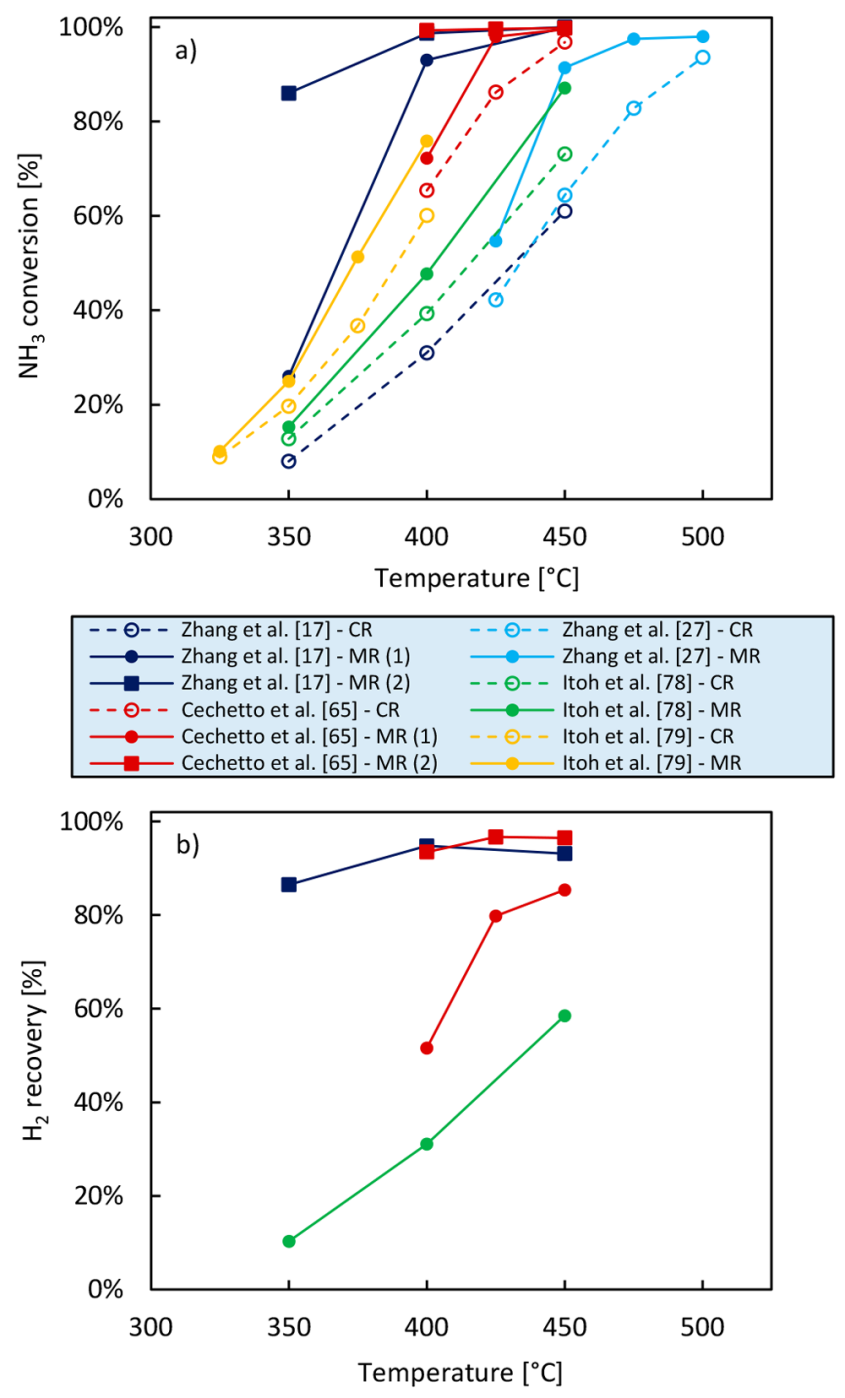
Comparison between the NH_3_ conversion (a) and H_2_ recovery (b) achieved with a conventional reactor (CR) and with a membrane reactor (MR) for ammonia decomposition in the works of Zhang et al.,^17^ Itoh et al.,^79,80^ Cechetto et al.,^65^ and Zhang et al.^27^

In Figure 5(a) and Table 3, the NH_3_ conversion achieved in the works of Zhang et al.,^17^ Itoh et al.,^79,80^ Cechetto et al.,^65^ and Zhang et al.^27^ both in membrane and in conventional reactors is depicted as a function of the operating temperature. It is possible to observe that in the conventional configuration the conversion is limited, especially at low temperatures, and does not reach the thermodynamic equilibrium. On the other hand, the conversion significantly increases when the membrane reactor is adopted. Operating the permeate at atmospheric pressure, Cechetto et al.^65^ performed ammonia decomposition at 4 bar over a Ru/Al_2_O_3_ catalyst and achieved an NH_3_ conversion higher than the conventional equilibrium conversion from 425 °C. By further applying vacuum at the permeate side of the membrane, i.e., favoring hydrogen removal from the reaction zone, NH_3_ conversion significantly overcomes the equilibrium conversion even at 400 °C. Similarly, Zhang et al.^17^ performed ammonia decomposition at 5 bar and with the membrane permeate at atmospheric pressure, obtaining NH_3_ conversion higher than the equilibrium conversion from 450 °C when Ru was impregnated in the membrane support and from 400 °C when Cs was used as a promoter.

**Table 3:** Comparison between NH_3_ Conversion and H_2_ Recovery Achieved with a Conventional Reactor (CR) and with a Membrane Reactor (MR) for Ammonia Decomposition in the Works of Zhang et al.,^17^ Itoh et al.,^79,80^ Cechetto et al,.^65^ and Zhang et al.^27^

Author(s) [ref]	Permeate pressure [bar]	Permeate pressure [bar]	NH_3_ flow rate [mL_N_/min]	Catalyst	Temperature [°C]	Equilibrium conversion [%]	NH_3_ conversion conventional reactor [%]	NH_3_ conversion membrane reactor [%]	Percentage increase in NH_3_ conversion	H_2_ recovery [%]
Zhang et al.^17^	5	1	20–100	1) Ru (Ru impregnated in the membrane support) (0.41 wt % Ru loading)	350	91.9	8	26	+225%	N/A
					400	95.9	31	93	+200%	N/A
					450	97.8	61^a^	∼100^a^	+64%	N/A
				2) Cs/Ru (Ru impregnated in the membrane support and Cs impregnated in the Pd layer)(Cs/Ru molar ratio = 1.5)	350	91.9	8	86	+975%	87
					400	95.9	31	99^a^	+219%	95
					450	97.8	61^a^	∼100^a^	+64%	93
										
Zhang et al.^27^	5	1	200	(Ni/Al = 1.20, La/Ni = 0.22) Ni/La-Al_2_O_3_, 6 g	425	97.0	42^a^	55^a^	+30%	N/A
					450	97.8	64^a^	91^a^	+42%	N/A
					475	98.3	83^a^	98^a^	+18%	N/A
					500	98.7	94^a^	98^a^	+5%	N/A
										
Cechetto et al.^65^	4	1) 1	500	(2 wt %) Ru/Al_2_O_3_, 250 g	400	96.7	65.4	72.2	+10%	94
					425	97.6	86.2	98.0	+14%	97
					450	98.2	96.8	99.5	+3%	97
		2) 0.01			400	96.7	65.4	99.3	+52%	52
					425	97.6	86.2	99.6	+16%	80
					450	98.2	96.8	99.8	+3%	85
										
Itoh et al.^79^	1	0.01	8.5–10	(5 wt %) Ru/SiO_2_, 0.5 g	350	98.2	13^a^	15^a^	+20%	10
					400	99.1	39^a^	48^a^	+21%	31
					450	99.7	73^a^	87^a^	+19%	59
										
Itoh et al.^80^	1	0.06	10	(2 wt %) Ru/Al_2_O_3_, 0.88 g	325	97.3	9^a^	10^a^	+13%	N/A
					350	98.2	20^a^	25^a^	+27%	N/A
					375	98.7	37^a^	51^a^	+40%	N/A
					400	99.1	60^a^	76^a^	+26%	N/A

a Data not directly reported in the publication and retrieved from graphic representation of experimental results.

Generally, the benefits in terms of NH_3_ conversion introduced with the use of a membrane reactor are more pronounced at lower temperatures. The lower the temperature is, in fact, the more ammonia decomposition is both thermodynamically and kinetically limited, and consequently, the more a kinetic enhancement and equilibrium shift would be beneficial. However, from Figure 5(a) and Table 3, it is possible to notice that in some works this trend is not verified and that NH_3_ conversion at low temperature is significantly lower compared to the equilibrium conversion—although still higher than conversion in a conventional reactor. Zhang et al.^17^ ascribed this limitation to the choice of a catalyst with insufficient activity at such low temperature. By impregnating the catalytic Pd membrane layer with Cs, NH_3_ conversion at 350 °C increased from 26% to 86%. Similarly, the relatively low NH_3_ conversion achieved by Zhang et al.^27^ at 425 °C compared to the one achieved at the same temperature by Cechetto et al.^40^ might be due to the lower catalytic activity of Ni compared to Ru. Itoh et al.^79,80^ attributed the low conversion achieved in their work to the high thickness of the selective layers of their membranes hindering a sufficient hydrogen removal. They therefore concluded that thinner membranes could be used to improve NH_3_ conversion especially at low temperatures. In reality, the enhancement level given by the membrane reactor strongly depends on the ratio between the membrane area installed (and thus the hydrogen flux) and the ammonia feed flow. The higher this ratio is, the larger the enhancement is as also indicated for other systems.^90^ Following these results, it is clear that only a good combination of operating conditions, optimal membrane, and catalyst selection can make the membrane reactor advantageous compared to a conventional system. It is worth noting that the possibility to achieve very high conversion at temperatures below 400 °C is very interesting because on one hand the energy efficiency is greatly improved, and on the other hand, the energy required to drive the ammonia decomposition can be easily supplied from flue gas in the downstream hydrogen utilization equipment. Figure 5(b) and Table 3 report also the available data of hydrogen recovery achieved in the same works for which NH_3_ conversion has been depicted in Figure 5(a) as a function of temperature. Further discussion in the following sections is addressed at explaining how the relevant performance indicators of a membrane reactor are affected by the selection of different operating conditions.

### 4.3 Effect of Membrane Reactor Operating Conditions on NH_3_ Conversion, H_2_ Recovery, and H_2_ Purity

An efficient membrane reactor design requires the selection of optimal operating conditions allowing for high ammonia conversion and high hydrogen recovery, while at the same time ensuring compactness and stability of the system as well as good energy efficiency/management. Since a good understanding of how the operating conditions of the system impact on the performance of the membrane reactor is paramount for an optimized reactor design, in this section, the effects of reaction temperature, reaction pressure, ammonia feed flow rate, and pressure at the permeate side of the membrane are explored. Particularly, an explanation on the effect of the selection of different operating conditions on NH_3_ conversion, H_2_ recovery, and H_2_ purity is given by analyzing experimental evidence available in the literature.

#### 4.3.1 Effect of Temperature

**Figure 6:**
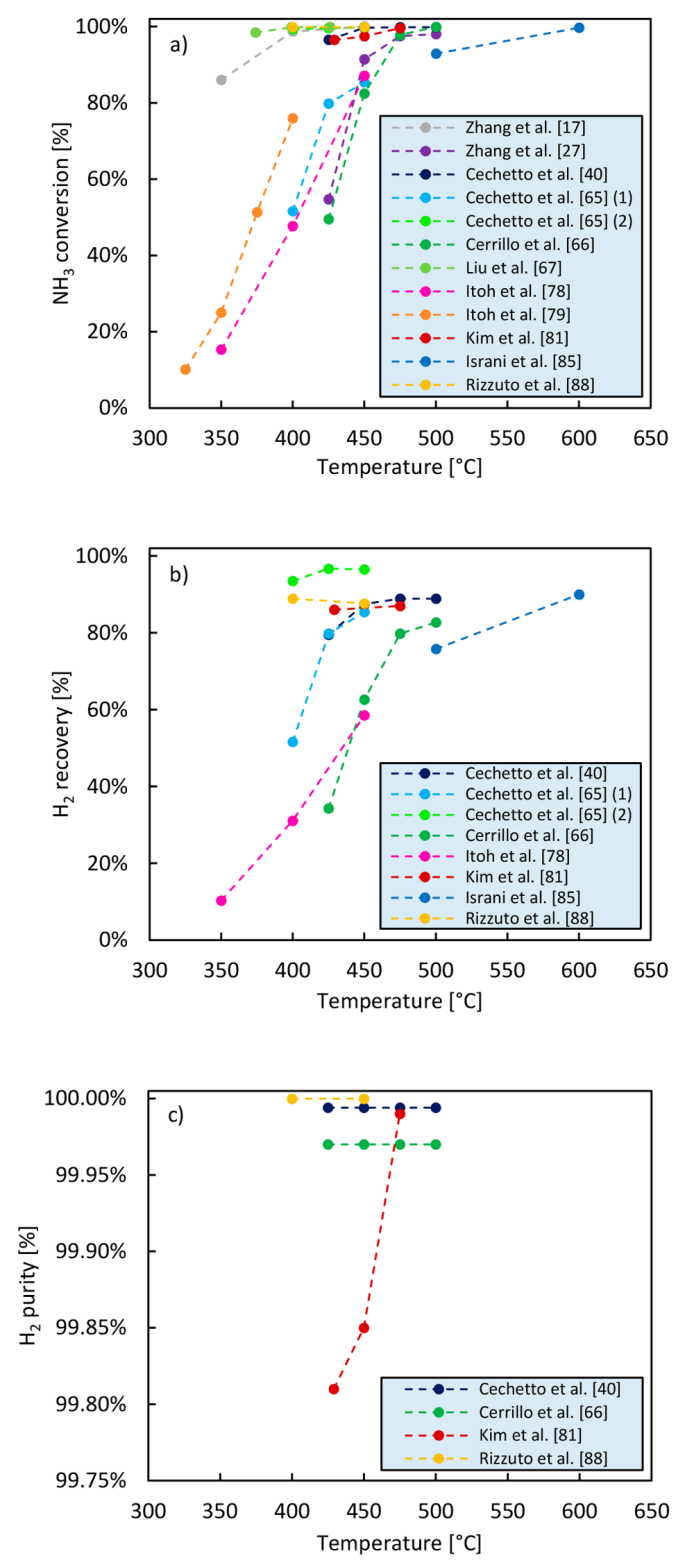
Effect of the ammonia decomposition reaction temperature on NH_3_ conversion (a), H_2_ recovery (b), and H_2_ purity (c) achieved in a membrane reactor. The data reported in this figure have been retrieved from studies available in the literature.

The results of relevant studies available in the literature in which the effect of temperature has been investigated are depicted in Figure 6. In Table 4, the operating conditions and the type of catalyst used in these works are reported. In general terms, the selection of a high operating temperature is beneficial as it results in high ammonia conversion and high recovery as well as high hydrogen purity. This can be explained based on the following considerations:

- Ammonia decomposition is an endothermic reaction; therefore, ammonia conversion into hydrogen and nitrogen is favored at high temperatures.
- The above-mentioned increase in conversion results in higher hydrogen partial pressure in the reactor, leading to a high driving force for hydrogen separation and therefore to higher H_2_ recovery.
- Higher conversion results in lower ammonia concentration in the retentate and, consequently, lower driving force for ammonia permeation through the membrane and higher hydrogen purity in the permeate.
- With the exception of membranes based on elements of group V such as Nd, V, and Ta, the permeability of hydrogen selective membranes generally increases with increasing temperature. High temperature therefore results into high hydrogen recovery.

**Table 4:** Operating Conditions for Data in Figure 6

Author(s) [ref]	Reaction pressure [bar]	Permeate pressure [bar]	NH_3_ flow rate [mL_N_/min]	Membrane selective layer composition	Catalyst
Zhang et al.^17^	5	1	20–100	Pd	Ru (impregnated in the membrane support)
Zhang et al.^27^	5	1	200	Pd	(Ni/Al = 1.20, La/Ni = 0.22) Ni/La-Al_2_O_3_, 6 g
Cechetto et al.^40^	4	1	500	Pd-Ag	(2 wt %) Ru/Al_2_O_3_, 250 g
Cechetto et al.^65^	4	1) 1	500	Pd-Ag	(2 wt %) Ru/Al_2_O_3_, 250 g
		2) 0.01			
Cerrillo et al.^66^	5	1	200	Pd-Au	(5 wt %) Ba-CoCe, 10 g
Liu et al.^67^	3	1	50	SiO_2_	(5 wt %) Ru/MgO, 1.5 g
Itoh et al.^79^	1	0.01	8.5–10	Pd	(5 wt %) Ru/SiO_2_, 0.5 g
Itoh et al.^80^	1	0.06	10	1) Pd	(2 wt %) Ru/Al_2_O_3_, 0.88 g
				2) Pd-Ag	
Kim et al.^82^	4	Vacuum	N/A	Pd	(2 wt %) Ru/Al_2_O_3_, N/A
Israni et al.^86^	5	1	200	Pd (nanopore)	(70 wt %) Ni/γ-Al_2_O_3_, 36 g in PBR and 29 g in PBMR
Rizzuto et al.^89^	5	1	N/A	Pd	(N/A) Ru/Al_2_O_3_, N/A

However, some trade-off has to be taken into account when selecting the optimal operating temperature considering the following:

- Membrane stability can decrease at high temperatures. Pd-based membranes suffer in fact from deterioration phenomena when exposed to temperatures higher than 500 °C, and therefore this temperature should never be exceeded.
- Low temperature should be targeted to improve the energy efficiency, lower the operation costs, and maximize the beneficial increase in performance compared to the conventional reactor.

#### 4.3.2 Effect of Reaction Pressure

**Figure 7:**
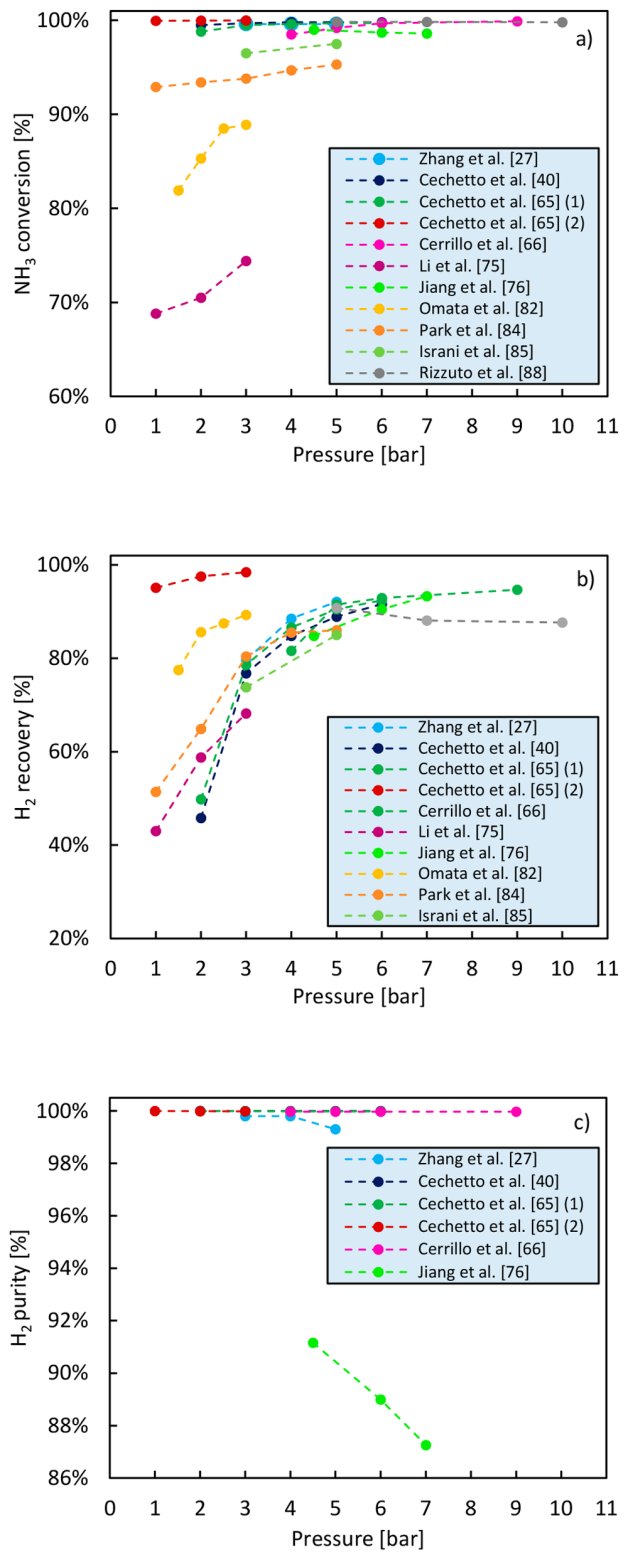
Effect of the ammonia decomposition reaction pressure on NH_3_ conversion (a), H_2_ recovery (b), and H_2_ purity (c) achieved in a membrane reactor. The data reported in this figure have been retrieved from studies available in the literature.

The results of studies available in the literature in which the effect of reaction pressure on the performance of a membrane reactor for ammonia decomposition has been investigated are presented in Figure 7, and the operating conditions and the type of catalyst used in these works are reported Table 5. The results of these studies demonstrate that the reactor operating pressure should be selected taking into account the following considerations:

- A sufficiently high pressure is required in a membrane reactor in order to provide driving force for separation and thus ensure a good hydrogen recovery.
- Increased hydrogen removal from the reaction zone at higher retentate pressure results in faster kinetics and shifted thermodynamics which in turn enhance ammonia conversion counterbalancing the ammonia conversion decrease that would on the other hand thermodynamically be expected according to the Le Châtelier’s principle.
- High reaction pressure negatively affects hydrogen purity: the higher the reaction pressure is, in fact, the higher is the concentration of impurities in the permeate, namely, N_2_ and NH_3_, due to the increased driving force for separation.

**Table 5:** Operating Conditions for Data in Figure 7

Author(s) [ref]	Temperature [°C]	Permeate pressure [bar]	NH_3_ flow rate [mL_N_/min]	Membrane selective layer composition	Catalyst
Zhang et al.^27^	500	1	200	Pd	(Ni/Al = 1.20, La/Ni = 0.22) Ni/La-Al_2_O_3_, 6 g
Cechetto et al.^40^	500	1	500	Pd-Ag	(2 wt %) Ru/Al_2_O_3_, 250 g
Cechetto et al.^65^	450	1) 1	500	Pd-Ag	(2 wt %) Ru/Al_2_O_3_, 250 g
		2) 0.01			
Cerrillo et al.^66^	485	1	200	Pd-Au	(5 wt %) Ba-CoCe, 10 g
Li et al.^76^	450	0.5	40	SiO_2_	(0.45 wt %) Ru/γ-Al_2_O_3_/α-Al_2_O_3_, Ru impregnated in the membrane support
Jiang et al.^77^	450	1	10	MFI zeolite	(3 wt %) Ru/Y/K/Al_2_O_3_, 1 g
Omata et al.^83^	350	1	10	Pd-Ag/V-Fe	(5 wt %) Ru/Cs_2_O/Pr_5_O_11_, 0.2 g
Park et al.^85^	500	1 (100 mL/min of steam as sweep gas)	100	Pd/Ta/Pd	(0.65 wt %) Ru/(10 mol %) La-Al_2_O_3_, 1 g
Israni et al.^86^	500	1	126	Pd	(70 wt %) Ni/γ-Al_2_O_3_, 29 g
Rizzuto et al.^89^	450	1	245	Pd	(N/A) Ru/Al_2_O_3_, N/A

All in all, the reactor operating pressure should be selected as a trade-off between the pressure allowing the maximization of hydrogen recovery and the one guaranteeing the targeted high hydrogen purity.

#### 4.3.3 Effect of Permeate Pressure

Atmospheric pressure or vacuum conditions at the permeate side of the membrane allow one to minimize the partial pressure of hydrogen at the permeate side of the membrane, increasing the driving force for its separation. Cechetto et al.^65^ experimentally compared the performance of a membrane reactor for ammonia decomposition with the permeate at atmospheric conditions and the performance achieved upon vacuum application. It was demonstrated that the application of vacuum enhances the performance of the membrane reactor both in terms of conversion and hydrogen recovery. While performing ammonia decomposition at 400 °C, 4 bar, and under a feed flow rate of 500 mL_N_/min of pure ammonia, NH_3_ conversion in fact increased from 72.2% to 99.3% subsequent to vacuum application, while the thermodynamic equilibrium conversion for these specific operating conditions (without membrane) is calculated to be 96.7%. Itoh et al.,^79,80^ Kim et al.,^82^ and Li et al.^76^ also performed ammonia decomposition in a membrane reactor with the permeate side at vacuum conditions. The results achieved in these studies are summarized in Table 6.

**Table 6:** Overview of Performance Achieved When Operating a Membrane Reactor with the Permeate at Vacuum Conditions

Author(s) [ref]	Temperature [°C]	Reaction pressure [bar]	NH_3_ feed flow rate [mL_N_/min]	Space velocity [mL_N_/(g_cat_ h)]	NH_3_ conversion [%]	H_2_ recovery [%]
Cechetto et al.^65^	400	4	500	120	99.3	93.5
Li et al.^76^	400	1	10	N/A	84	77
Itoh et al.^79^	450	1	10	1200	87^a^	59^a^
Itoh et al.^80^	400	1	10	682	76^a^	N/A
Kim et al.^82^	430	5	950	N/A	99.4	97.5

a Data not directly reported in the publication and retrieved from graphic representation of experimental results.

**Figure 8:**
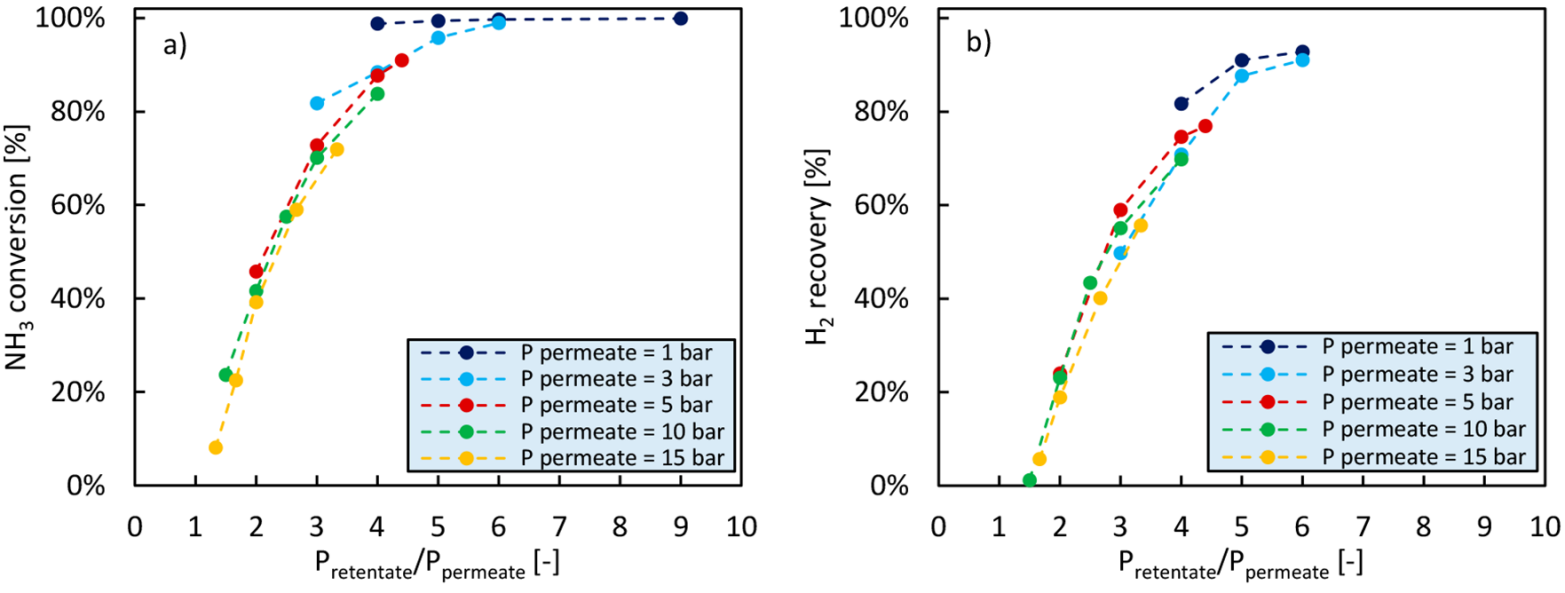
Influence of permeate pressure for different retentate pressures on NH_3_ conversion (a) and H_2_ recovery (b). The experimental data presented in this figure have been retrieved from the work of Cerrillo et al.^66^ The experimental results have been obtained performing the decomposition of 200 mL_N_/min of pure ammonia at 485 °C on a catalyst bed consisting of 10 g of 0.5% Ba-CoCe diluted with 10 g of SiC.

Pressurized hydrogen can be required for some applications, requiring the addition of a compression step to the process if hydrogen is produced at ambient or vacuum pressure. As a pressurized permeate would allow one to reduce or avoid this cost, Cerrillo et al.^66^ investigated the production of hydrogen from ammonia decomposition at a pressure higher than atmospheric. Specifically, they performed ammonia decomposition at 485 °C varying the pressure at the permeate side of the membrane from 1 to 15 bar, while increasing the pressure in the retentate chamber from 4 to 50 bar. The main experimental results of this study have been retrieved from their publication and are presented in Figure 8, in which NH_3_ conversion and H_2_ recovery are represented as a function of the ratio between the pressures at the retentate and permeate sides of the membrane, respectively. As expected, NH_3_ conversion and H_2_ recovery decrease with decreasing P_retentate_/P_permeate_. This makes it clear that operating the reactor with a pressurized permeate is not really desirable when targeting the production of ultrapure (NH_3_-free) hydrogen. While this study might therefore not be relevant for PEMFC application as their optimal operating pressure typically lies between 3 and 4 bar,^91^ the results achieved in this work interestingly demonstrate that the membrane reactor for ammonia decomposition has a great degree of flexibility.

#### 4.3.4 Effect of NH_3_ Feed Flow Rate

**Figure 9:**
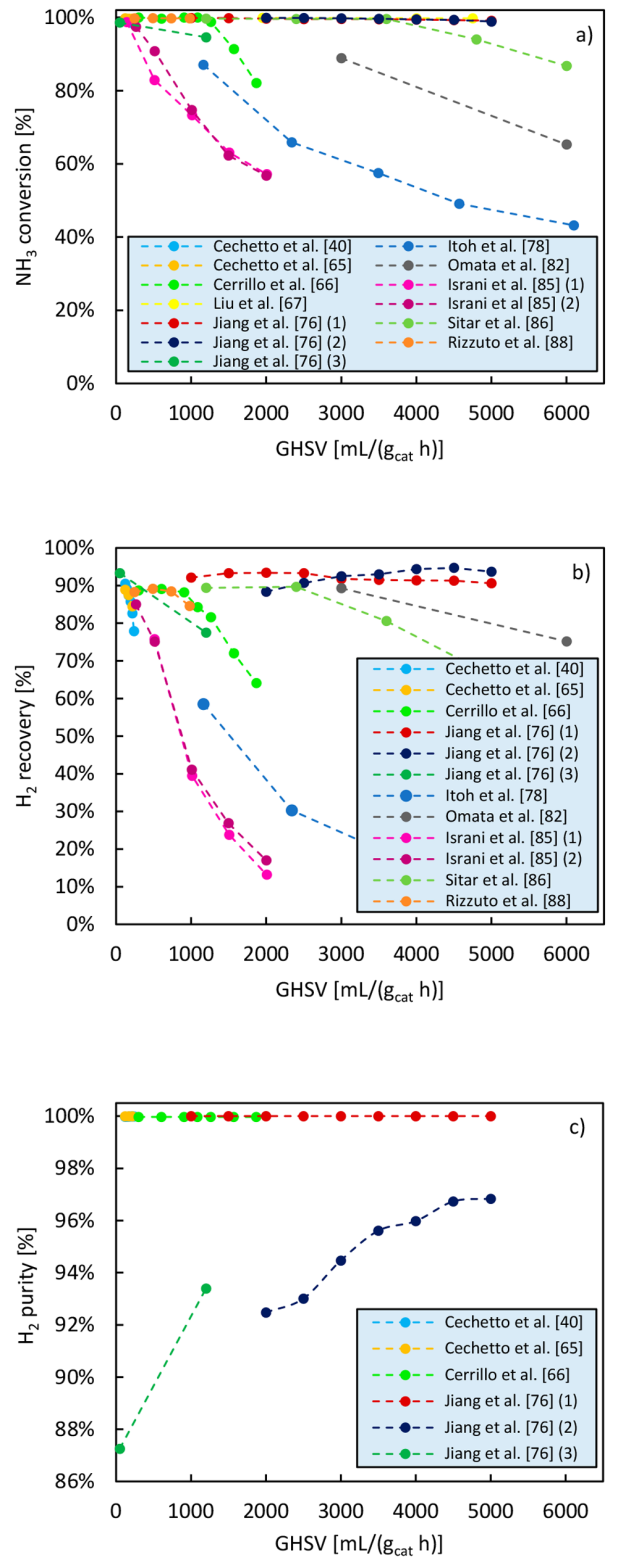
Effect of ammonia feed flow rate on NH_3_ conversion (a), H_2_ recovery (b), and H_2_ purity (c) achieved in a membrane reactor. The data reported in this figure have been retrieved from studies available in the literature.

The effect of NH_3_ feed flow rate on the membrane reactor performance is depicted in Figure 9 based on literature data. In Table 7, the operating conditions and the type of catalyst used in these works are reported. As ammonia feed flow rate increases, the residence time in the reactor decreases, and this results in lower NH_3_ conversion and lower hydrogen recovery. However, the produced hydrogen purity increases. These results show that high ammonia feed flow rates can be considered if high H_2_ purity at the outlet of the reactor is targeted. However, the recycle of unreacted NH_3_ and residual H_2_ in the retentate—or their possible integration as heat sources to sustain the endothermic system in the overall energy balance—should in this case be taken into account in the overall plant design.

**Table 7:** Operating Conditions for Data in Figure 9

Author(s) [ref]	Temperature [°C]	Reaction pressure [bar]	Permeate pressure [bar]	Membrane selective layer composition	Catalyst
Cechetto et al.^40^	500	5	1	Pd-Ag	(2 wt %) Ru/Al_2_O_3_, 250 g
Cechetto et al.^65^	450	5	1	Pd-Ag	(2 wt %) Ru/Al_2_O_3_, 250 g
Cerrillo et al.^66^	485	4	1	Pd-Au	(0.5 wt %) Ba-CoCe, 10 g
Liu et al.^67^	425	3	1	Pd/Pd-Ag	(5 wt %) Ru/MgO, 1.5 g
Jiang et al.^77^	450	7	1	1) Pd-Ag	1) (3 wt %) Ru/Y/K/Al_2_O_3_, 3 g
				2) CMSM	2) (3 wt %) Ru/Y/K/Al_2_O_3_, 3 g
				3) MFI zeolite	3) (3 wt %) Ru/Y/K/Al_2_O_3_, 1 g
Itoh et al.^79^	450	1	0.01	Pd	(5 wt %) Ru/SiO_2_, 0.5 g
Kim et al.^82^	430	5	Vacuum	Pd	(2 wt %) Ru/Al_2_O_3_, N/A
Omata et al.^83^	350	3	1	Pd-Ag/V-Fe	(5 wt %) Ru/Cs_2_O/Pr_5_O_11_, 0.2 g
Israni et al.^86^	500	5	1) 1	Pd (nanopore)	(70 wt %) Ni/γ-Al_2_O_3_, 29 g
			2) Pd		
Sitar et al.^87^	450	5	1	Pd	(0.5 wt %) Ru/Al_2_O_3_, 5 g in the catalyst bed
					(1.9 wt %) Ru/YSZ, Ru impregnated in the membrane support
Rizzuto et al.^89^	450	10	1	Pd	Ru/Al_2_O_3_, N/A

#### 4.3.5 Efficient Membrane Reactors for Hydrogen Production from Ammonia Decomposition

In this section, the results that document the best performance achieved in membrane reactors for ammonia decomposition available in literature are shown. To this end, we define efficient membrane reactors as reactors in which a conversion of NH_3_ >99% and an H_2_ recovery >90% are simultaneously achieved. Accordingly, the results of the studies in which efficient membrane reactor operation was achieved are reported in Table 8. Next to the NH_3_ conversion and H_2_ recovery achieved, information on the reactor operating conditions and the catalyst and membrane used as well as on the volumetric hydrogen productivity and the total catalyst utilization is also reported. The volumetric hydrogen productivity and the total catalyst utilization, when not indicated in the publication, have been calculated from the properties and performance values of both the membrane and the reactor reported. Specifically, they have been calculated as the molar flow rate of hydrogen produced via ammonia decomposition in the membrane reactor per unit volume of the reactor and as the amount of catalyst in the reactor per unit volume of the membrane, respectively. Ammonia conversion >99% can be generally achieved at a nominally standard operating temperature of 450 °C and pressures between 5 and 7 bar if the reactor is operated with the permeate side of the membrane at atmospheric conditions using a Ru-based catalyst, whereas higher temperature is required with less active catalysts. Hydrogen recovery and hydrogen productivity are on the other hand dependent on the ammonia feed flow rate and on the membrane reactor separation performance. The best performance was achieved by Jiang et al.,^77^ who reported the highest volumetric productivity while efficiently using a highly active catalyst. The excellent performance of their system was achieved thanks to the use of both a highly efficient catalyst and a membrane with a low thickness for the selective layer (1.8 μm) which allowed for high hydrogen permeation. Despite a higher catalyst loading, Cechetto et al.^65^ achieved the lowest volumetric hydrogen productivity, which can be attributed to the reduced hydrogen permeance due to the high thickness of the membrane selective layer. Cerrillo et al.^66^ achieved the best performance in terms of catalyst utilization, but no information is available for the calculation of the volumetric hydrogen productivity achieved in their work. Given the high thickness of the membrane selective layer, we expect it however to be lower compared to the one achieved by Jiang et al.^77^

**Table 8:** Performance of Efficient Membrane Reactors (NH_3_ Conversion >99; H_2_ Recovery >90%) for Hydrogen Production from Ammonia Decomposition

Author(s) [ref]	Catalyst loading [wt %] (catalyst)	Membrane type and thickness [μm]	Temperature [°C]	Pressure [bar]	Pressure permeaten [bar]	NH_3_ conversion [%]	H_2_ recovery [%]	Volumetric productivity [mol m^–3^ s^–1^]	Total catalyst utilization [g/cm^2^]
Zhang et al.^17^	0.41 (Cs-Ru/YSZ)	Pd, 6.2	450	5	1	>99	–	31.6	N/A
Cechetto et al.^40^	2 (Ru/Al_2_O_3_)	Pd-Ag, 6–8	500	6	1	99.8	91.6	1.2^a^	2.915^a^
Cechetto et al.^65^	2 (Ru/Al_2_O_3_)	Pd-Ag, 4.61	450	5	1	99.7	90.5	1.2^a^	2.992^a^
			400	4	Vacuum	99.3	93.5	1.3^a^	2.814^a^
Cerrillo et al.^66^	5 (Ba-CoCe)	Pd-Au, 8	485	5	1	99^a^	91^a^	N/A	0.143
Jiang et al.^77^	3 (Ru-Y-K/Al_2_O_3_)	Pd-Ag, 1.8	450	7	1	>99.1	>90.6	>40.8^a^	0.168^a^
Kim et al.^82^	2 (Ru/Al_2_O_3_)	Pd, 5	430	5	Vacuum	99.4	97.5	1.5^a^	N/A
Sitar et al.^87^	1.9 (Ru/YSZ)	Pd, 4.23	450	5	1	>99	>90	54.2	0.221
	0.5 (Ru/α-Al_2_O_3_)								

a Not directly reported in the publication. Calculated based on provided membrane/reactor properties and performance values.

For completeness of this article, a summary of the performance achieved in all the articles that have been reviewed is reported in Table 9. The table includes information about the configuration of the membrane used for hydrogen separation, operating conditions of the membrane reactor, catalyst employed, and the best results achieved in each work. For best results, we consider the results that allowed the achievement of efficient membrane reactor operation (NH_3_ conversion >99% combined with H_2_ recovery >90%) or, when efficient reactor operation was not achieved, the results that were the closest to this target.

**Table 9:** NH_3_ Conversion and Corresponding Hydrogen Recovery Achieved in Membrane Reactors for Ammonia Decomposition Studies Available in Literature

	Membrane			Reactor operating conditions						NH_3_ conversion [%]		
Author(s) [ref]	Membrane composition	Selective layer thickness [μm]	Length [mm]	Temperature [°C]	Reaction pressure [bar]	Permeate pressure [bar]	NH_3_ feed flow rate [mL_N_/min]	GHSV [mL/(g_cat_ h)]	Catalyst	Conventional reactor	Membrane reactor	Hydrogen recovery [%]
Zhang et al.^17^	Pd + Ru/YSZ	6.2	N/A	400	5	1	30^a^	N/A	Ru (impregnated in the membrane support)	31^a^	93	N/A
	Ru-Cs/YSZ + Pd			400	5	1	61.3	N/A		N/A	98	87.5
Zhang et al.^27^	Pd + Al_2_O_3_	3	N/A	500	5	1	200^b^	2000	Ni/La-Al_2_O_3_ (Ni/Al = 1.20, La/Ni = 0.22), 6 g	95^a^	>99^a^	92^a^
Cechetto et al.^40^	Al_2_O_3-_–YSZ + Pd-Ag + Al_2_O_3_	∼6–8	195	500	6	1	500	120^b^	(2 wt %) Ru/Al_2_O_3_, 250 g	N/A	99.8	91.6
Cechetto et al.^65^	Al_2_O_3_-YSZ + Pd-Ag + Al_2_O_3_	4.61	202	400	4	Vacuum	500	120^b^	(2 wt %) Ru/Al_2_O_3_, 250 g	65.4	99.3	93.5
			190	450	5	1	500	120^b^	(2 wt %) Ru/Al_2_O_3_, 250 g	N/A	99.7	90.5
Cerrillo et al.^66^	Pd-Au + ceramic support	8	186^a^	485	5	1	200	1200^b^	(0.5 wt %) Ba-CoCe, 10 g	N/A	>99^a^	92^a^
Liu et al.^67^	Pd + stainless steel support + MnCO_3_	6.5	N/A	400	3	1	47^b^	1880	(5 wt %) Ru/MgO, 1.5 g	N/A	99.8	N/A
Li et al.^76^	Ru/γ-Al_2_O_3_/α-Al_2_O_3_+ SiO_2_	<0.3	N/A	400	1	Vacuum	10	N/A	(0.45 wt %) Ru/γ-Al_2_O_3_/α-Al_2_O_3_, Ru impregnated in the membrane support	55	84	77
Jiang et al.^77^	Pd-Ag + Al_2_O_3_	1.8	100^a^	450	7	1	250^b^	5000	(3 wt %) Ru/(1 wt %)Y/(12 wt %) K/Al_2_O_3_, 3 g	N/A	99.11	90.6
Itoh et al.^79^	Pd	200	65	450	1	Vacuum	9.7	1164^b^	(5 wt %) Ru/SiO_2_, 0.5 g	73^a^	87^a^	59^a^
Itoh et al.^80^	Pd + Al_2_O_3_ + Ru	2	90	375	1	Vacuum	20	N/A	(2 wt %) Ru/Al_2_O_3_, 0.88 g	35^a^	>99^a^	N/A
Kim et al.^82^	Pd + Inconel 600	∼5	450	430	5	Vacuum	950	N/A	(2 wt %) Ru/Al_2_O_3_, N/A	N/A	99.4	97.5
Omata et al.^83^	Pd-Ag + V-Fe	∼0.2 μm Pd-Ag	N/A	350	3	1	10	3000^b^	(5 wt %) Ru/Cs_2_O/Pr_6_O_11_, 0.2 g	52.6%^a^	89^a^	89^a^
		∼100 μm V-Fe										
Jo et al.^84^	Pd/Ta	∼0.4 μm Pd	N/A	450	6.5	1	3000^b^	30000	(1.6 wt %) Ru/La-Al_2_O_3_, 6 g	N/A	>99.5	N/A
		∼250 μm Ta										
Park et al.^85^	Pd/Ta/Pd	∼1–2 μm Pd	N/A	500	5	1	100^b^	6000	(0.65 wt %)Ru/(10 mol %)La-Al_2_O_3_, 1 g	N/A	95^a^	86^a^
		∼250 μm Ta										
Israni et al.^86^	Pd/γ-Al_2_O_3_/α-Al_2_O_3_	∼13	156	500	3	1	65^a^	135^b^	(70 wt %) Ni/γ-Al_2_O_3_, 29 g	N/A	99^a^	80^a^
Sitar et al.^87^	Pd + Ru/YSZ	4.23	73	450	5	1	100	1200^b^	(0.5 wt %) Ru/Al_2_O_3_, 5 g in the catalyst bed	N/A	>99^a^	>90^a^
									(1.9 wt %) Ru/YSZ, Ru impregnated in the membrane support			
Rizzuto et al.^89^	Pd + metallic support	N/A	N/A	450	5	1	245	N/A	(N/A) Ru/Al_2_O_3_, N/A	N/A	>99^a^	91^a^

a Data not directly reported in the publication and retrieved from graphic representation of experimental results.

b Data not directly reported in the publication. Calculated based on provided information about catalyst and flow rates used.

#### 4.3.6 Hydrogen Purity and Residual Ammonia Traces

As previously mentioned, PEMFC specifications impose that hydrogen used as feedstock must contain a maximum residual NH_3_ concentration of 0.1 ppm. In case hydrogen is produced via ammonia decomposition, monitoring the purity of hydrogen produced and, particularly, its residual ammonia content is therefore paramount to prevent fuel cell electrodes poisoning. The experimental studies performed with membrane reactors for ammonia decomposition reporting information about the purity of hydrogen produced are compiled in Table 10, in which for each work the membrane reactor characteristics, the type of catalyst used, and the reaction conditions are reported next to the information regarding the purity of hydrogen produced and the residual ammonia concentration in the hydrogen stream. The best performances in terms of hydrogen purity have been achieved in membrane reactors implementing Pd-based membranes, and this is due to the fact that, compared to other type of membranes, Pd-based membranes show outstanding performance in terms of selectivity toward hydrogen separation.^72,92^ This was demonstrated by Jiang et al.^77^ who performed ammonia decomposition over a Ru/Y/K/Al_2_O_3_ catalyst and compared the performance of different types of membranes, namely, a modified MFI zeolite membrane, a carbon molecular sieve membrane, and a Pd-Ag membrane. While with all these membranes it was possible to obtain both NH_3_ conversion >99% and H_2_ recovery >90% under pressurized NH_3_ feed of 7 bar, the purity of hydrogen produced as well as the residual NH_3_ concentration in the hydrogen stream were demonstrated to be highly influenced by the different separation performances of the membranes. Hydrogen conforming to the ISO standard for fuel cell vehicle application—i.e., with ammonia and nitrogen concentrations less than 0.1 and 300 ppm, respectively—was produced when performing the decomposition of an ammonia feed flow rate ranging between 50 and 250 mL/min over 3 g of a Ru/Y/K/Al_2_O_3_ catalyst at 450 °C and with the the membrane reactor implementing a Pd-Ag membrane with a 1.8 μm thick selective layer. More specifically, by means of a gas chromatographer and an NH_3_ sensor for ammonia concentrations in the range between 10 ppb and 50 ppm, the N_2_ and NH_3_ concentrations in the hydrogen stream were measured to be below 0.01% and 10 ppb, respectively. On the other hand, when the reactor was equipped with a CMSM and with a modified MFI zeolite membrane, the poorer separation performance compared to the Pd-Ag membrane led to hydrogen purities <97% and <94%, respectively, which are therefore not compatible for a correct functioning of a PEMFC. Similarly, Omata et al.^83^ produced hydrogen conforming to the ISO standard for fuel cell vehicle application by performing the decomposition of 10 mL/min of pure ammonia over a Ru/Cs_2_O/Pr_6_O_11_ at 350 °C and 3 bar in a membrane reactor implementing a V-Fe/Pd-Ag membrane with a 100 μm thick V-Fe selective layer and 250 thick Pd-Ag selective layer. The residual ammonia concentration was estimated to be 0.06 ppm from the measurement carried out with an ion chromatograph on the amount of ammonium ion contained in 30 mL of a sulfuric acid aqueous solution bubbled with the hydrogen produced in the membrane reactor. Compared to the results achieved by Jiang et al.,^77^ in this work, fuel cell-grade hydrogen could be produced at significatively lower temperature. This has an advantage in terms of energy efficiency of the system. It is however worth noticing that the achievement of this result was only possible thanks to the use of a significantly thicker, thus more expensive, membrane.

**Table 10:** Studies Related to Ammonia Decomposition in Membrane Reactors Reporting the Purity of Hydrogen Produced and Residual Ammonia Concentration in the Hydrogen Stream

	Membrane			Reactor operating conditions						Hydrogen purity		
Author(s) [ref]	Selective layer composition	Selective layer thickness [μm]	Length [mm]	Temperature [°C]	Reaction pressure [bar]	Permeate pressure [bar]	NH_3_ feed flow rate [mL_N_/min]	GHSV [mL/(g^–1^_cat_ h)]	Catalyst type	Maximum H_2_ purity [%]	NH_3_ concentration in the permeate [ppm]	Method used for NH_3_ concentration measurement
Zhang et al.^17^	Pd	6.2	N/A	450	5	1	100	N/A^a^	Cs (0.41 wt %) Ru/YSZ, Ru impregnated in the membrane support	>99.7	<1000	NDIR spectroscopy
Zhang et al.^27^	Pd	∼3	N/A	500	3	1	400	4000^b^	Ni/La-Al_2_O_3_ (Ni/Al = 1.20, La/Ni = 0.22), 6 g	>99.9^a^	N/A	N/A
Cechetto et al.^40^	Pd-Ag	∼6–8	195	500	2	1	500	120^b^	(2 wt %) Ru/Al_2_O_3_, 250 g	99.998	<0.75	FTIR spectroscopy
Cechetto et al.^65^	Pd-Ag	4.61	202	450	1	Vacuum	500	120^b^	(2 wt %) Ru/Al_2_O_3_, 250 g	99.995	2.91	FTIR spectroscopy
Cerrillo et al.^66^	Pd-Au	8	N/A	350–500	4–15	1	100–310	600–1870^b^	(0.5 wt %) Ba-CoCe, 10 g	99.97 ± 0.03	N/A	Gas chromatography
Liu et al.^67^	Pd/Pd-Ag	6.5	N/A	400	3	1	47	1880^b^	(5 wt %) Ru/MgO, 1.5 g	99.85^a^	N/A	N/A
Li et al.^76^	SiO_2_	<0.3	N/A	400	1	Vacuum	10	N/A	(0.45 wt %) Ru/γ-Al_2_O_3_/α-Al_2_O_3_, Ru impregnated in the membrane support	84.0	45,000	Gas chromatography
Jiang et al.^77^	MFI zeolite	8	N/A	450	4.5	1	10	600	(3 wt %) Ru/(1 wt %) Y/(12 wt %) K/Al_2_O_3_, 3 g	91.16	3500	Gas chromatography
	CMSM	0.9	220	450	7	1	250	5000		96.84	<10,000	Gas chromatography and NH_3_ sensor
	Pd-Ag	1.8	80	450	7	1	50–250^b^	1000–5000		>99.999	<0.01	
Omata et al.^83^	Pd-Ag/V-Fe	0.2/100	N/A	350	3	Vacuum	10	3000^b^	(5 wt %) Ru/Cs_2_O/Pr_6_O_11_, 0.2 g	<99.9975	<0.06	Ion chromatography
Jo et al.^84^	Pd/Ta	∼0.4 μm Pd	N/A	450	6.5	1	3000^b^	30000	(1.6 wt %) Ru/La-Al_2_O_3_, 6 g	>99.9999	0.8	Tunable diode laser spectrometry
		∼250 μm Ta										
Park et al.^85^	Pd/Ta/Pa	∼1–2	76	425	3	1	20^b^	1200	(0.65 wt %) Ru/(10 mol %)La-Al_2_O_3_, 1 g	N/A	4.2	IR spectroscopy
Sitar et al.^87^	Pd	4.23	73	450	5	1	100–300	1200–3600^b^	(0.5 wt %) Ru/Al_2_O_3_, 5 g in the catalyst bed	N/A	650^a^	Draeger tube
									(1.9 wt %) Ru/YSZ (Ru impregnated in the membrane support)			

a Data not directly reported in the publication and retrieved from graphic representation of experimental results.

b Data not directly reported in the publication. Calculated based on provided information about catalyst and flow rates used.

In other works, despite a purity of hydrogen higher than 99% being achieved, the compliance with the residual NH_3_ limit imposed by the specifications on PEMFCs was challenging. Cechetto et al.,^65^ for instance, while obtaining a purity of hydrogen of 99.995% measured the concentration of NH_3_ in the stream to be 2.9 ppm through FTIR measurements. This result was achieved at 450 °C, with a pressure difference across the membrane of 1 bar, under a feed flow rate of 500 mL_N_/min and with a membrane with a 4.61 μm thick selective layer. In a follow up work, Cechetto et al.^40^ demonstrated that a targeted hydrogen purity can be achieved by properly tuning the reactor operating conditions as well as increasing the thickness of the membrane selective layer. Under a similar pressure difference across the membrane and NH_3_ feed flow rate compared to their previous work, Cechetto et al. demonstrated the production of 99.998% pure hydrogen containing an NH_3_ concentration lower than the FTIR accuracy limit of 0.75 ppm at 500 °C with a membrane having an ∼6–8 μm thick selective layer. Cerrillo et al.^66^ performed ammonia decomposition over a Co-based catalyst in a packed bed membrane reactor using a Pd-Au alloy membrane with 8 μm thick selective layer and achieved purities higher than 99.97 ± 0.03% for temperature ranging between 350 and 500 °C, pressures between 4 and 15 bar, and a NH_3_ feed flow rate ranging between 100 and 310 mL_N_/min of pure ammonia. Moreover, Cerrillo et al.^66^ report that the hydrogen produced in this system is NH_3_ free and could therefore directly be used as feedstock for PEMFCs. While this seems to be a very promising result, the authors report that residual ammonia concentrations were measured by means of a micro-GC of which however the detection limits are unknown.

As is possible to see from Table 10, in most of the works, the purity of hydrogen produced did not meet the requirements for fuel cell application. However, while hydrogen produced in these works is not suitable to be directly used as fuel for such devices, it could be still used for this purpose if a hydrogen purification stage would be introduced between the membrane reactor and the PEMFC. Several studies available in the literature demonstrate in fact that the purification of hydrogen from residual ammonia can be carried out by means of commercially available NH_3_ sorbents as well as by means of zeolites.^44−47,93^ Sitar et al.^87^ experimentally demonstrated that the residual ammonia impurities in the hydrogen stream can be reduced from ∼1000 ppm to values below 0.025 ppm using the earth-abundant zeolite clinoptilolite as adsorbent material for ammonia removal. The measure of such low NH_3_ concentrations was determined by analyzing the position of the reaction front and the volume of gas passing through Draeger tubes packed with a yellow adsorbent material that irreversibly turns purple upon ammonia exposure. In agreement with the results of this study, Cechetto et al.^40^ experimentally demonstrated that with the addition of a hydrogen purification unit consisting of a bed of zeolite 13X at ambient conditions it is possible to produce hydrogen with residual ammonia concentration below 0.75 ppm even at 450 °C and using a membrane with an ∼1 μm thick selective layer. Although this solution makes the system more complex, the benefits introduced in the system are such that this solution might be regarded as the most favorable both from an economic and an energy point of view. The introduction of a hydrogen purification stage offers in fact the possibility to produce ultrapure hydrogen while adopting a lower reactor temperature as well as thinner membranes, lowering energy consumption and costs.

Li et al.^76^ performed ammonia decomposition in a silica-based catalytic membrane reactor and reported that at 400 °C and under a NH_3_ feed flow rate of 10 mL/min the purity and the NH_3_ concentration in the permeate stream were 84.0% and 4.5%, respectively. While these results suggest that the purity of hydrogen achieved with this type of reactor is significantly lower compared to the purity achievable in a Pd-based membrane reactor and not sufficiently high for direct hydrogen utilization in PEMFC, the authors believe that that the advantages of a silica-based catalytic membrane reactor may compensate for the fact that in order to obtain fuel cell-grade hydrogen a hydrogen purification unit downstream the reactor should be implemented increasing the system complexity.

Kim et al.^82^ investigated hydrogen production by ammonia decomposition over a Ru/Al_2_O_3_ catalyst using a Pd-based membrane on porous metallic support with a selective layer thickness of ∼1 μm. While they report that at 472 °C, 5 bar, NH_3_ feed flow rate of 2840 mL/min, and with the permeate side of the membrane at vacuum conditions the production of 99.99% pure hydrogen could be achieved, the purity of hydrogen was measured through gas chromatography downstream a water trap and a cold trap that served to eliminate the residual traces of ammonia in the stream.

Finally, Jo et al.^84^ and Park et al.^85^ investigated a membrane reactor for hydrogen production from ammonia decomposition integrated with a PEMFC. In both these works, despite containing traces of ammonia, the hydrogen produced via ammonia decomposition in the membrane reactor was fed to a PEMFC, and in the work of Jo et al.,^84^ no degradation of the device was observed for an extended time of operation. Jo et al. demonstrated that performing ammonia decomposition over a Ru/La-Al_2_O_3_ catalyst in a packed bed membrane reactor using a dense metallic Pd/Ta composite membrane with a 0.4 μm thick Pd selective layer and 250 μm thick Ta selective layer it is possible to produce hydrogen with purities higher than 99.9999% and residual ammonia concentration of 0.8 ppm (measured by Nessler method) when working at 450 °C, 6.5 bar, and with an ammonia feed flow rate of 30,000 mL/g^–1^_cat_ h^–1^. Park et al.^85^ produced hydrogen containing a residual ammonia concentration of 4.2 ppm (measured by Nessler method) while performing ammonia decomposition at 425 °C, 3 bar, and with an ammonia feed flow rate of 1200 mL/g^–1^_cat_ h^–1^ in a membrane reactor coupling a custom developed Pd/Ta composite metallic membrane and a ruthenium on lanthanum-doped alumina catalyst (Ru (0.65 wt. %)/La (10 mol. %)-Al_2_O_3_). In both works, steam was adopted as the sweep gas allowing for separation of pure hydrogen upon its condensation. The condensed water could be subsequently recirculated in the system prior to vaporization exploiting the waste heat from the system and serve as an NH_3_ scrubber in the hydrogen stream. Park et al.^85^ demonstrated that the amount of ammonia in the permeated hydrogen could be reduced from 4.2 to 1.8 ppm after flowing the hydrogen stream through a gas–liquid separator containing 300 cm^3^ of bubbling DI water.

## 5 Membrane Reactor for Ammonia Decomposition in Diluted Streams

Even though this review focuses on the production of PEM fuel cell-grade hydrogen from pure ammonia, for the sake of completeness, in this section, we report the works that have investigated the use of a membrane reactor for ammonia decomposition as cleaning technology in diluted NH_3_ streams.

As a matter of fact, the earliest studies on membrane reactors for ammonia decomposition addressed the gas cleaning of streams containing NH_3_ traces. Collins et al.,^94^ for example, suggested the use of a membrane reactor as a strategy to reduce the trace amount of ammonia from coal gasification processes which are responsible for nitrogen oxide (NO_*x*_) emissions and experimentally investigated the potential of this technology using a simplified gas composition of H_2_, N_2_, and NH_3_ in typical concentrations of an integrated gasification combined cycle (IGCC) synthesis. A composite palladium-ceramic membrane with thickness of the selective layer of 11.4 μm and 55 mm active lengths and a supported Ni/Al_2_O_3_ catalyst were used for hydrogen separation and as the catalyst, respectively. It was demonstrated that the membrane reactor can significantly improve ammonia conversion when diluted concentrations of ammonia and high concentrations of hydrogen and nitrogen are fed to the membrane reactor. Similarly, other studies investigated NH_3_ decomposition in membrane reactors considering feed streams in which ammonia is very diluted, such as purge streams from ammonia production plants or synthesis gas streams from coal gasification plants.^95−100^ Although interesting from a fundamental perspective, these studies investigate ammonia decomposition at different operating conditions and reaction kinetics compared to the ones in which ammonia is regarded as a hydrogen carrier for hydrogen production for PEM fuel cell applications, and their results are thus not extensively reviewed in this article.

Moreover, we chose not to include in this review article the results of studies in which nitrogen, helium, or other noble gases have been used at the permeate side of the membrane as sweep gases.^25,88,101^ In fact, while the use of a sweep gas favors hydrogen recovery, the hydrogen produced with this technique is diluted and therefore not interesting for pure hydrogen production purposes. Articles in which steam has been use as the sweep gas^85^ are on the other hand reviewed, since although apparently diluting the produced hydrogen stream, steam can be easily separated from hydrogen via condensation resulting in the production of pure hydrogen. A summary of experimental results available in the literature on membrane reactors for ammonia decomposition using diluted streams as feed and/or sweep gas at the permeate side of the membrane is presented in Table 11.

**Table 11:** Summary of Literature Available on Experimental Studies on Membrane Reactors for Decomposition of Ammonia Available in Diluted Streams or Sweep Gas

	Membrane				Reactor operating conditions								
Author(s) [ref]	Configuration	Selective layer composition	Selective layer thickness [μm]	Length [mm]	Temperature [°C]	Reaction pressure [bar]	Permeate pressure [bar]	Feed [mL_N_/min]	Feed composition [mol %]	Sweep gas flow rate [mL/min]	Catalyst type	Highest NH_3_ conversion [%]	Hydrogen recovery [%]
Li et al.^25^	Silica membrane on bimodal catalytic support (Ru/γ-Al_2_O_3_/α-Al_2_O_3_)	SiO_2_	<0.0003	N/A	450	1	1	10	100 (NH_3_)	100 (N_2_)	Ru	94.3	93^a^
Li et al.^88^	Silica membrane on bimodal catalytic support (Ru/γ-Al_2_O_3_/α-Al_2_O_3)_	SiO_2_	<0.0003	N/A	390–470	1	1	10–50	100 (NH_3_)	10–100 (N_2_)	Ru	96^a^	91^a^
Collins et al.^94^	Supported composite Pd-ceramic membranes	Pd	11.4	55	450–600^a^	16.18	1	435	48 (N_2_)	N/A	Ni/Al_2_O_3_	>94	N/A
									20 (H_2_)				
									31.665 (He)				
									0.335 (NH_3_)				
García-García et al.^95^	Pd/porous stainless steel composite membrane	Pd	40	N/A	333–592^a^	1	1	100	90 (He)	100–300 (He)	Carbon supported Ru-based catalyst promoted with sodium (2 wt % Ru)	100	N/A
									10 (NH_3_)				
Cheng et al.^101^	Dual layer NMW/NMW-Ni hollow fiber membrane	Nd_5.5_Mo_0.5_W_0.5_O_11.25-δ_ (NMW)	26	N/A	600–750	1	1	50	60–80 mol % He	50 mL/min N_2_	NMW-NiO catalytic support of the membrane (0.11 g)	99	N/A
									20–40 mol % NH_3_				

a Data not directly reported in the publication and retrieved from graphic representation of experimental results.

## 6 Challenges and Perspectives

Storing hydrogen in the form of liquid ammonia offers a promising solution to the current obstacles associated with physical storage and distribution of hydrogen, which hinder the progress of the hydrogen economy. Additionally, due to its lack of carbon emissions, ammonia could serve as a cleaner substitute for fossil fuels, playing a crucial role in the decarbonization of the current energy system. Nevertheless, the implementation of an ammonia-based energy system necessitates further research to tackle numerous challenges, spanning the entire value chain of ammonia production, distribution, and application.

First, ammonia can be regarded as a cost-effective CO_2_-free energy and hydrogen carrier only under the assumption that ammonia production and utilization processes are powered by renewable energy and do not imply direct or indirect CO_2_ emissions.^31^ Future research needs therefore to be addressed in the attempt to decarbonize the ammonia production step as at the moment more than 90% of the global production of ammonia are carried out in the conventional Haber–Bosch process.^102^ The Haber–Bosch process produces ammonia at relatively high temperatures and pressures in the ranges of 400–500 °C and 10–30 MPa, with an energy consumption ranging between 28 to 37 GJ/*t*_NH_3__.^103^ Moreover, since it generally uses as feedstock for ammonia synthesis hydrogen produced from fossil fuels (coal, natural gas, naphtha, or oil), the ammonia production process is responsible for carbon dioxide emissions ranging between 2.0 and 4.6 *t*_CO_2__/*t*_NH_3__,^104^ for a total annual emission of about 400 Mt_CO_2__, which represents around 1.5% of all greenhouse gas emissions.^102^ The decarbonization of this process should therefore tackle several challenges among which are the production of green ammonia with a cost competitive to fossil fuel-derived ammonia and the design of a system capable of being flexible enough to follow the fluctuations and intermittency of renewable electricity sources.^105^ Pursuing this mission, Hydrogen Utility (H2U) in partnership with ThyssenKrupp has developed The Port Lincoln Hydrogen Energy Storage System (Eyre Peninsula, South Australia, Australia), one of the first commercial plants capable of producing green ammonia from intermittent energy resources. This demonstration plant includes a 30 MW water electrolysis plant, an ammonia production facility with a capacity of 50 tons per day, and two 16 MW open-cycle turbines operating 100% on hydrogen at the site to provide electricity to the grid during periods of low wind or solar output.^106^ While H2U expects to sell the relatively small amount of green ammonia produced in this facility into the local agriculture market as fertilizer, this plant will demonstrate the techno-economic viability of the novel supply chain technology of green hydrogen in the form of ammonia.^106^

Other major obstacles toward the deployment of ammonia-based energy systems are then mainly related to the hydrogen production and purification steps through ammonia decomposition. Hydrogen production from ammonia is in fact an energy intensive process; thus, research into materials, technologies, and possible plant configurations is essential to achieve high energy efficiencies in the utilization of ammonia at different scales. Particularly, one of the main challenges consists of the development of a new generation of catalysts to replace the Ru-based ones, which while leading at the moment the highest reaction rate and the lowest temperature are on the other hand extremely expensive. The formulation of new catalysts should therefore target the reduction of the ammonia decomposition temperature as well as the use of readily available, non-noble materials, ensuring the reduction of both the operational and capital expenditures of the system, respectively. As far as the materials and technologies for hydrogen purification from ammonia are concerned, relatively inexpensive commercial adsorbent materials and water absorption units have been demonstrated to be effective, with little need for new technologies and materials development. Nevertheless, it is crucial to use accurate instruments with adequate detection limits to measure the purity of hydrogen produced, particularly for PEM fuel cell applications that require extremely low residual ammonia concentration. Similarly, precise analytical instrumentation should be used to measure the ammonia concentration in the hydrogen stream at the permeate side of membrane reactors, especially in situations in which the selectivity of membranes is high enough to potentially produce fuel cell-grade hydrogen without downstream hydrogen purification. Research on materials should finally explore new routes for the fabrication of economically competitive membranes with high selectivity toward hydrogen. Current literature suggests that Pd-based membranes can achieve a H_2_/N_2_ selectivity sufficiently high to eliminate the need for a N_2_ separation unit in the system, even in systems for hydrogen production for vehicle applications. However, the high investment cost associated with Pd-based membranes has to be assessed and necessitates the development of cheaper formulations capable of providing comparable separation properties comparable.

An additional technical aspect worthy of attention pertains to the mode of heat generation which is required to sustain the ammonia decomposition reaction. To promote the development of a sustainable and environmentally friendly energy system, heating powered by renewables or green fuels should be preferred over conventional carbon-based fuels. The direct combustion of ammonia is economically favorable, but involves the generation of a significant amount of harmful nitrogen oxides (NO_*x*_). Selective catalytic reduction (SCR) or selective noncatalytic reduction (SNCR) processes, as well as stoichiometric controlled oxidation (SCO) technology, can be used to achieve in a single unit both complete NH_3_ combustion and limited NO_*x*_ formation.

While in the literature it has been demonstrated that from a technical point of view the production of fuel cell-grade hydrogen in a membrane reactor-based system is possible regardless of the type of membrane when using a hydrogen purification stage downstream the membrane reactor, very little information is available regarding the economic feasibility of the system. Yet, some studies calculated the costs of hydrogen production from ammonia decomposition,^31,39^ but to the best of our knowledge, a comparative study addressing a techno-economic assessment at different plant capacities and configurations is not available. Additional work should therefore investigate the techno-economic feasibility of an NH_3_-to-H_2_ system, identifying the design choices and plant capacities that make a membrane reactor-based system more competitive compared to a conventional system. Techno-economic feasibility studies should investigate both centralized plants in which hydrogen is produced in large quantities remotely and is subsequently transported to the point of end use, as well as decentralized scenarios in which hydrogen is produced using smaller-scale production systems to supply local demand. The difference in plant capacity as well as in the location relative to the end point of use of hydrogen have in fact an impact on the final cost of the technology which varies on a case-by-case basis.

## 7 Conclusions

In this review article, we have reported the latest developments in membrane reactors for hydrogen production from ammonia decomposition, especially looking at operating conditions, membrane properties, and possible downstream separation/purification units. All works report remarkable improvements in terms of hydrogen production and ammonia conversion compared to conventional systems, and the following few general considerations can be drawn:

- Membrane reactors allow working at lower temperatures and higher pressures compared to conventional systems with an increase of energy efficiency. Ammonia is transported as liquid at higher pressures (>8 bar at least), and this means that high pressure can be easily used in membrane reactors to achieve high conversion at a limited membrane area. Ideally, the membrane reactor should be operated at the same pressure of the ammonia storage to avoid additional compressions in between.
- As the membrane reactor can work at lower temperatures compared to conventional systems, temperatures below 500 °C are preferable. By optimizing catalysts and membrane flux (and increasing the installed membrane area), the reactor could even be operated at temperatures lower than 400 °C, which would allow easier heat integration of the reactor with the upstream and downstream apparatuses.
- The very important parameters to be optimized in the ammonia decomposition unit are hydrogen recovery and hydrogen purity, while conversions lower than 100% are not a problem as the retentate stream can be used to generate the energy required for the vaporization of the feed and the heating of the reactor.
- According to the literature, the best performing membranes seem to be Pd-based ones. However, fuel cell-grade hydrogen could only be achieved with relatively thick membranes. On the other hand, the latest results also show that relatively inexpensive sorption units downstream of the membrane reactor can allow to easily achieve fuel cell-grade hydrogen production (containing NH_3_ concentration <0.1 ppm, for the detection of which, dedicated and accurate analytical systems are key). These results show that fuel cell-grade hydrogen can also be produced working with thinner membranes, thus improving the CAPEX of the system, or even using much less expensive membranes such as ceramic or carbon membranes.
